# A Donor–Acceptor‐Type Two‐Dimensional Poly(Arylene Vinylene) for Efficient Electron Transport and Sensitive Chemiresistors

**DOI:** 10.1002/anie.202504302

**Published:** 2025-05-02

**Authors:** Ruyan Zhao, Wei Wang, Yamei Liu, Petko Petkov, Arafat Hossain Khan, Lei Gao, Peng Zhang, Eike Brunner, Hai I. Wang, Shivam Singh, Shirong Huang, Luis Antonio Panes‐Ruiz, Yana Vaynzof, Mischa Bonn, Gianaurelio Cuniberti, Mingchao Wang, Xinliang Feng

**Affiliations:** ^1^ Max Planck Institute of Microstructure Physics Weinberg 2 Halle 06120 Germany; ^2^ Faculty of Chemistry and Food Chemistry & Center for Advanced Electronics Dresden (cfaed) Technische Universität Dresden Mommsenstraße 4 Dresden 01062 Germany; ^3^ Institute for Materials Science and Max Bergmann Center for Biomaterials TUD Dresden Technische Universität Dresden Dresden 01062 Germany; ^4^ Faculty of Chemistry and Pharmacy Sofia University, St. Kliment Ohridski Sofia 1164 Bulgaria; ^5^ Max Planck Institute for Polymer Research Ackermannweg 10 Mainz 55128 Germany; ^6^ Nanophotonics Debye Institute for Nanomaterials Science Utrecht University Princetonplein 1 Utrecht 3584 CC The Netherlands; ^7^ Chair for Emerging Electronic Technologies Technische Universität Dresden Nöthnitzer Straße 61 Dresden 01187 Germany; ^8^ Leibniz Institute for Solid State and Materials Research Dresden Helmholtzstraße 20 Dresden 01069 Germany; ^9^ State Key Laboratory of Advanced Waterproof Materials School of Advanced Materials Peking University, Shenzhen Graduate School Shenzhen 518055 China

**Keywords:** 2D Poly(arylene vinylene), Chemiresistors, Covalent organic frameworks, Donor–acceptor, Electron transport

## Abstract

Two‐dimensional (2D) conjugated polymers and their layer‐stacked 2D conjugated covalent organic frameworks, such as 2D poly(arylene vinylene)s (2D PAVs), are emerging as promising polymer semiconductors for electronics and photocatalysis. However, achieving narrow optical band gaps and efficient electron transport remains a significant challenge for this class of materials to enhance the device's performance. Here, we report a donor‐acceptor‐type 2D PAV (**2DPAV‐TBDT‐IT**, where TBDT = thienyl‐benzodithiophene and IT = *s*‐indacene‐1,3,5,7(2*H*,6*H*)‐tetraone) synthesized via an Aldol‐type 2D polycondensation approach. Notably, **2DPAV‐TBDT‐IT** benefits from an effective intralayer donor–acceptor effect, exhibiting an optical band gap of 1.15 eV, the smallest among the reported 2D conjugated polymers. Density functional theory calculations reveal a unique electron‐dominating transport for **2DPAV‐TBDT‐IT**, with a strongly dispersive conduction band minimum and, thus, a small effective mass for electrons half that for holes. Additionally, terahertz spectroscopy measurements indicate a high charge mobility of 26 cm^2^ V^−1^ s^−1^ at room temperature for the powder sample. Given the high electron‐deficiency of **2DPAV‐TBDT‐IT** for facile electron injection from hazardous gases and the high‐mobility electron‐dominating transport in the material, we further fabricate chemiresistors from **2DPAV‐TBDT‐IT**, showing ultrasensitive SO_2_ analyte detection with limit of detection of 0.088 ppb, significantly surpassing the reported chemiresistive SO_2_ sensors.

## Graphical abstract

A donor–acceptor‐type two‐dimensional poly(arylene vinylene) has been developed, exhibiting a narrow optical band gap and a unique electron‐dominating transport with high charge carrier mobility toward ultrasensitive chemiresistors.

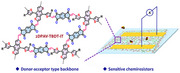

Two‐dimensional conjugated polymers (2D CPs) are represented by 2D conjugated covalent organic frameworks (2D c‐COFs), which present in‐plane extended π‐conjugation and out‐of‐plane electronic couplings.^[^ ^1^, ^2^, ^3^, ^4^, ^5^ ^]^ They are assembled from aromatic building blocks linked together by conjugated covalent linkages. Typical examples include C═N (e.g., imine, pyrazine)‐linked 2D c‐COFs^[^ ^6^, ^7^, ^8^, ^9^, ^10^, ^11^, ^12^ ^]^ (in which crystallinity relies on reversible or dynamic covalent chemistry^[^ ^13^, ^14^ ^]^), vinylene (or sp^2^‐carbon)‐linked 2D c‐COFs (also known as 2D poly(arylene vinylene)s, abbreviated as 2D PAVs),^[^ ^15^, ^16^ ^]^ and ladder‐type 2D poly(benzimidazobenzophenanthroline)s (known as 2D BBLs).^[^ ^17^, ^18^, ^19^ ^]^ In particular, 2D PAVs with robust polymeric skeletons have exhibited chemical stability and π‐conjugation efficiency significantly surpassing those of locally polarized C═N‐linked 2D c‐COFs.^[^ ^2^, ^20^, ^21^ ^]^ To date, various synthetic methodologies such as Knoevenagel,^[^ ^15^, ^16^, ^22^, ^23^ ^]^ Aldol‐type,^[^ ^24^, ^25^, ^26^, ^27^ ^]^ Horner–Wadsworth–Emmons,^[^ ^28^ ^]^ and Wittig^[^ ^29^ ^]^ polycondensation have been developed to construct crystalline 2D PAVs.

Efficient intralayer π‐conjugation and interlayer electronic coupling can confer remarkable electronic properties to 2D PAVs, making them promising candidates for electronics (e.g., transistors, chemiresistors, etc.), optoelectronics, photocatalysis, and photoelectrochemical catalysis.^[^ ^30^, ^31^, ^32^ ^]^ Considerable efforts have been dedicated to engineering the polymer backbone with electron‐rich molecular units such as pyrene or thiophene toward enhanced π‐conjugation and boosted charge carrier mobilities.^[^ ^16^, ^23^, ^33^, ^34^ ^]^ However, the currently developed 2D PAVs are yet unsatisfactory, showing optical band gaps generally wider than 1.6 eV, with holes being the dominant charge carriers. Inspired by the design strategy in linear conjugated polymers, deliberately bridging donor units by acceptor moieties can fine‐tune frontier‐orbital energy levels and reduce band gaps. Further tuning the electron deficiency of the acceptor units can lead to a strongly dispersive conduction band minimum (CBM), ultimately achieving *n*‐type semiconductors, which are indispensable for (opto)electronic applications, such as complementary logic circuits, chemiresistors, and solar cells. In this context, we envision that developing donor–acceptor‐type 2D PAVs with efficient conjugation would reduce the band gap and strengthen the dispersion of CBM to achieve efficient electron transport for future electronic applications.

In this work, we demonstrate the efficient synthesis of a novel donor–acceptor‐type 2D PAV (**2DPAV‐TBDT‐IT**; where the donor TBDT is thienyl‐benzodithiophene, and the acceptor IT is *s*‐indacene‐1,3,5,7(2*H*,6*H*)‐tetraone) through an Aldol‐type 2D polycondensation reaction. Benefiting from the effective intralayer donor–acceptor effect, **2DPAV‐TBDT‐IT** exhibits a narrow optical band gap of 1.15 eV, significantly lower than the donor–donor‐type **2DPAV‐TBDT‐BT** (1.60 eV; BT = bithiophene) and the reported 2D c‐COFs. Density functional theory (DFT) calculations of the band structure reveal a unique stronger dispersion in CBM (∼0.5 eV) compared to that in valence band maximum (VBM, ∼0.15 eV) for **2DPAV‐TBDT‐IT**. This distinctive band structure results in an effective mass for electrons (0.259 m_0_) that is nearly two times smaller than that for holes. This finding indicates an unprecedented electron‐dominating transport in **2DPAV‐TBDT‐IT**. Furthermore, ultrafast terahertz (THz) spectroscopy reveals a charge carrier mobility as high as 26 cm^2^ V^−1^ s^−1^ at room temperature for the powder sample. Given the high electron‐deficiency of **2DPAV‐TBDT‐IT** for facile electron injection from hazardous gases and the high‐mobility electron‐dominating transport in the material, we further fabricated chemiresistors from **2DPAV‐TBDT‐IT** showing ultrasensitive SO_2_ analyte detection at room temperature. The limit of detection is as low as 0.088 ppb, superior to the reported sensor materials.

TBDT represents an excellent *C*_2_‐symmetric building block for 2D PAVs.^[^ ^35^, ^36^ ^]^ To endow the polymer with efficient 2D conjugation, we performed DFT calculations on various TBDT‐based model compounds (Figure 1a). Compared to the TBDT/biphenyl‐based imine‐linked **M1** (Figures S1 and S2), the vinylene‐linked **M2** displays a slightly reduced HOMO‐LUMO (HOMO = highest occupied molecular orbital, LUMO = lowest unoccupied molecular orbital) energy gap of 2.93 eV (*vs*. 3.08 eV). Substituting biphenyl with the donor BT (**M3**) further enhances the *p*‐orbital interactions, resulting in a considerably diminished energy gap by 0.5 eV compared to **M2**. In comparison, the donor–acceptor‐type TBDT/IT‐based **M4** exhibits a slightly lower energy gap than **M3** (∼0.1 eV) but a lower‐lying LUMO extensively localized on the acceptor moieties (Figure 1b). This orbital configuration can favor the intramolecular charge transfer for narrow band‐gap semiconductors. By contrast, **M5** and **M6** display insufficient conjugation due to the energy mismatch between TBDT and the bipyridine/bipyrazine moieties.

**Figure 1:**
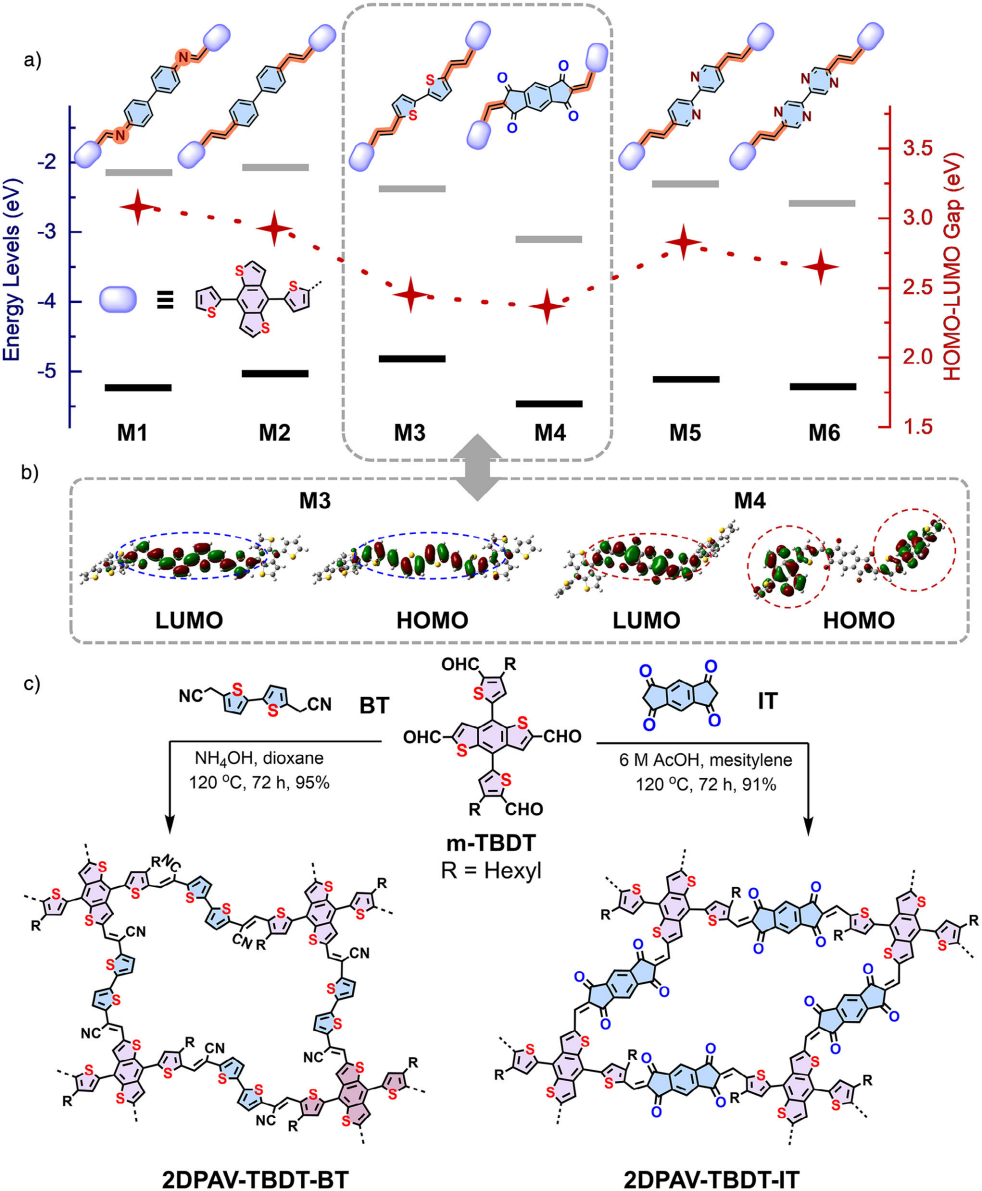
Design and synthesis of TBDT‐based 2D PAVs. a) Calculated energy levels of compound **M1–M6**. b) HOMO/LUMO of **M3** and **M4**. c) Synthetic routes of TBDT‐based 2D PAVs.

Encouraged by the above results, we developed the synthesis of the novel donor‐acceptor‐type **2DPAV‐TBDT‐IT** through an Aldol‐type 2D polycondensation between the 4,8‐bis(5‐formyl‐4‐hexylthiophen‐2‐yl)benzo[1,2‐*b*:4,5‐*b*']dithiophene‐2,6‐dicarbaldehyde (m‐TBDT) and IT monomers using acetic acid as the catalyst at 120 °C for 72 h (Figure 1c). To achieve the best crystallinity, different reaction conditions, including solvents, catalysts, temperatures, and reaction times, are screened and summarized in Table S1. Meanwhile, the donor‐donor‐type **2DPAV‐TBDT‐BT** was synthesized via a Knoevenagel 2D polycondensation of m‐TBDT and 2,2′‐([2,2′‐bithiophene]‐5,5′‐diyl)diacetonitrile using NH_4_OH as the catalyst at 120 °C for 72 h. The crystalline nature of **2DPAV‐TBDT‐IT** was resolved by powder X‐ray diffraction (pXRD) analysis, showing diffraction peaks at 5.41°, 7.54°, 10.75°, 15.32°, and 25.46°, which correspond to (001), (100), (10‐1), (212), and (‐114) reflections, respectively, for a slipped AA model (Figure 2a,b). Pawley refinement agrees well with the experimental result, as indicated by small *R*_wp_ value of 2.23% and *R*_p_ value of 1.43%. Moreover, the lattice parameters are obtained as *a* = 23.10 Å, *b* = 18.63 Å, *c* = 9.028 Å, *α* = 50.95°, *β* = 55.79°, *γ* = 80.10°. Scanning electron microscopy (SEM) images show that **2DPAV‐TBDT‐IT** appears as fibers aggregated into spherical shapes (Figure S3).

**Figure 2:**
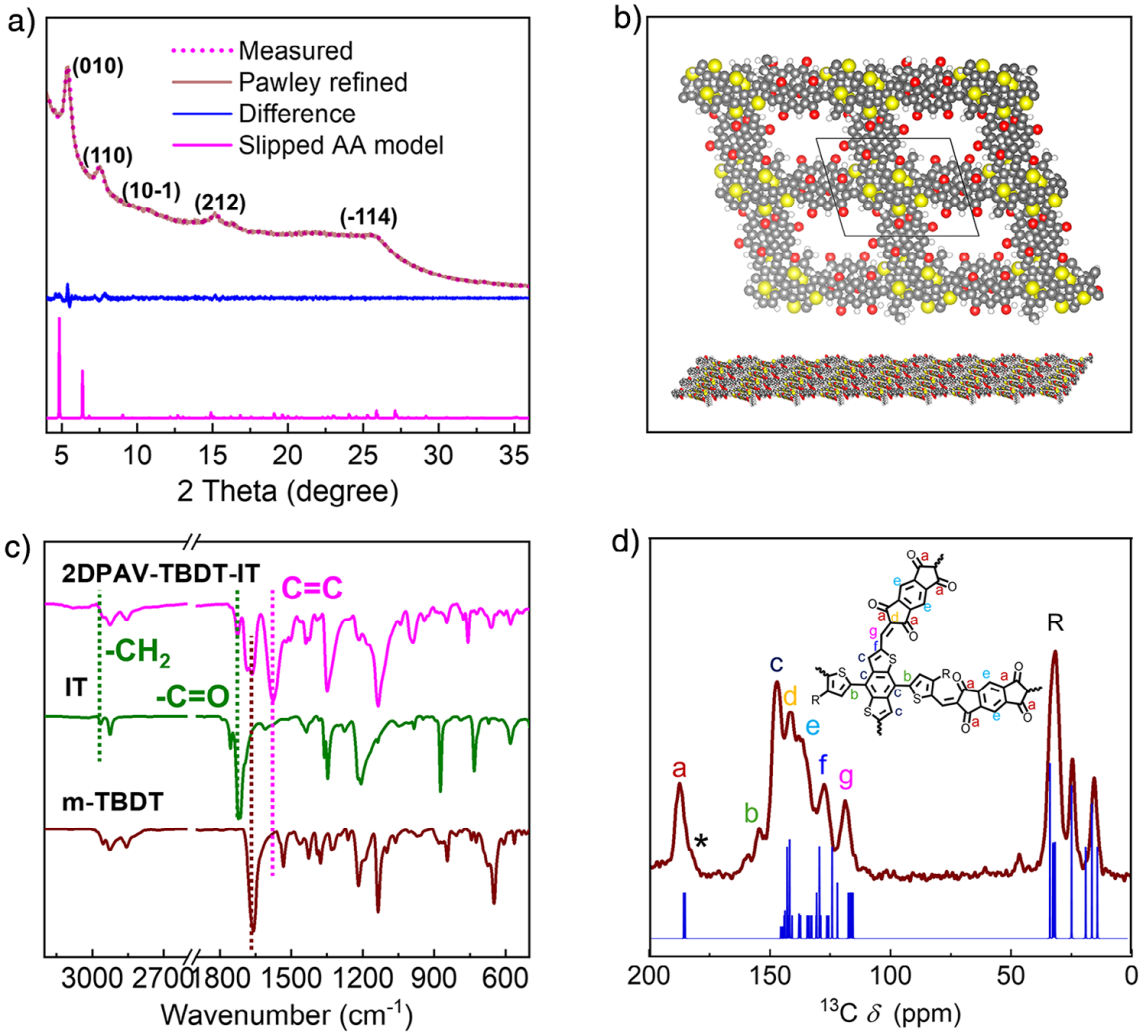
Characterizations of **2DPAV‐TBDT‐IT**. a) pXRD pattern. b) Multilayered model. Alkyl chains are not depicted. c) FT‐IR spectra. d) Experimental (red) and predicted (blue) ^13^C‐CP MAS NMR spectra.

Fourier‐transform infrared (FT‐IR) and solid‐state cross‐polarization magic angle spinning (CP MAS) nuclear magnetic resonance (NMR) spectroscopies were utilized to identify the chemical structure of **2DPAV‐TBDT‐IT**. As shown in the FT‐IR spectrum (Figure 2c and Figure S4), the disappearance of the characteristic methylene C─H and C═O stretching vibrations from the monomers, along with the presence of ─C═C─ stretching vibration at 1576 cm^−1^, indicate a successful formation of the vinylene linkages. Meanwhile, the ketone stretching vibrations of IT at 1755 and 1718 cm^−1^ are shifted to 1725 and 1686 cm^−1^, because of the extended π‐conjugation in the 2D PAV. Solid‐state depth ^1^H‐MAS NMR spectrum displays broad proton signals for **2DPAV‐TBDT‐IT** centered at 8 and 2 ppm, attributed to the aromatic and aliphatic protons, respectively. The proton signal presented above 10 ppm is attributed to the unreacted aldehyde groups that likely exist at the edges of the 2D PAV particles (Figure S5). In the CP ^13^C‐NMR spectrum (Figure 2d), three groups of distinct and well‐separated signals at 187, 110–170, and 10‒40 ppm are presented, corresponding to carbonyl groups, aromatic carbons, and alkyl chains, respectively. Two different signals of vinylene linkages appear at 141 (peak d) and 118 ppm (peak g), which are in good accordance with the predicted spectrum. The pXRD pattern, FT‐IR spectrum, and SEM image of **2DPAV‐TBDT‐BT** are shown in Figures S6–S8). Both **2DPAV‐TBDT‐BT** and **2DPAV‐TBDT‐IT** show superb thermal stability (Figures S9 and S10) and chemical stability in organic solvents and strong acidic/basic solutions (Figures S11‒S14). We also accessed their permanent porosities by nitrogen physisorption measurements (Figures S15–S18). The pore‐size distribution calculated by the nonlocal DFT method reveals an average pore size of 1.1 nm, which agrees well with the simulated structure model (Figures S15 and S16).

The energy band diagrams and the projected density of states (PDOS) are then calculated to explore the charge transport of the 2D PAV via the DFT/Perdew–Burke–Ernzerhof method (Figure 3a,b). The results suggest that the monolayer and the slipped‐AA‐stacked **2DPAV‐TBDT‐IT** are direct band‐gap semiconductors with a relatively flat VBM and a strongly dispersive CBM. From the PDOS, we observe hybridization of the C*p*, O*p*, and S*p* orbitals at VBM, while only the contribution of the C*p* and O*p* orbitals (from IT units) to the CBM. Taking monolayer **2DPAV‐TBDT‐IT** as an example, the bands show a stronger dispersion of ∼0.50 eV in the CBM and a relatively weaker dispersion of ∼0.15 eV in the VBM. To our best knowledge, such a unique band structure has not been observed for the reported 2D conjugated polymers and 2D c‐COFs.^[^ ^37^, ^38^ ^]^ This result implies that **2DPAV‐TBDT‐IT** should be an electron‐dominating transport material, i.e., an *n*‐type semiconductor. In contrast, **2DPAV‐TBDT‐BT** presents comparable band dispersions in VBM and CBM with no preferred charge carriers (Figure S20). We further calculated the electron density distribution of monolayer **2DPAV‐TBDT‐IT**. As depicted in Figure 3c, the VBM and CBM electron clouds are centered on TBDT and IT, respectively, revealing the intramolecular charge transfer from TBDT to IT via the vinylene linkages (Figure 3c). Similar to the monolayer, the slipped‐AA‐stacked **2DPAV‐TBDT‐IT** also presents a dispersive CBM and a relatively flat VBM, leading to a further decreased electron effective mass of 0.259 m_0_ and a large hole effective mass of 0.589 m_0_.

**Figure 3:**
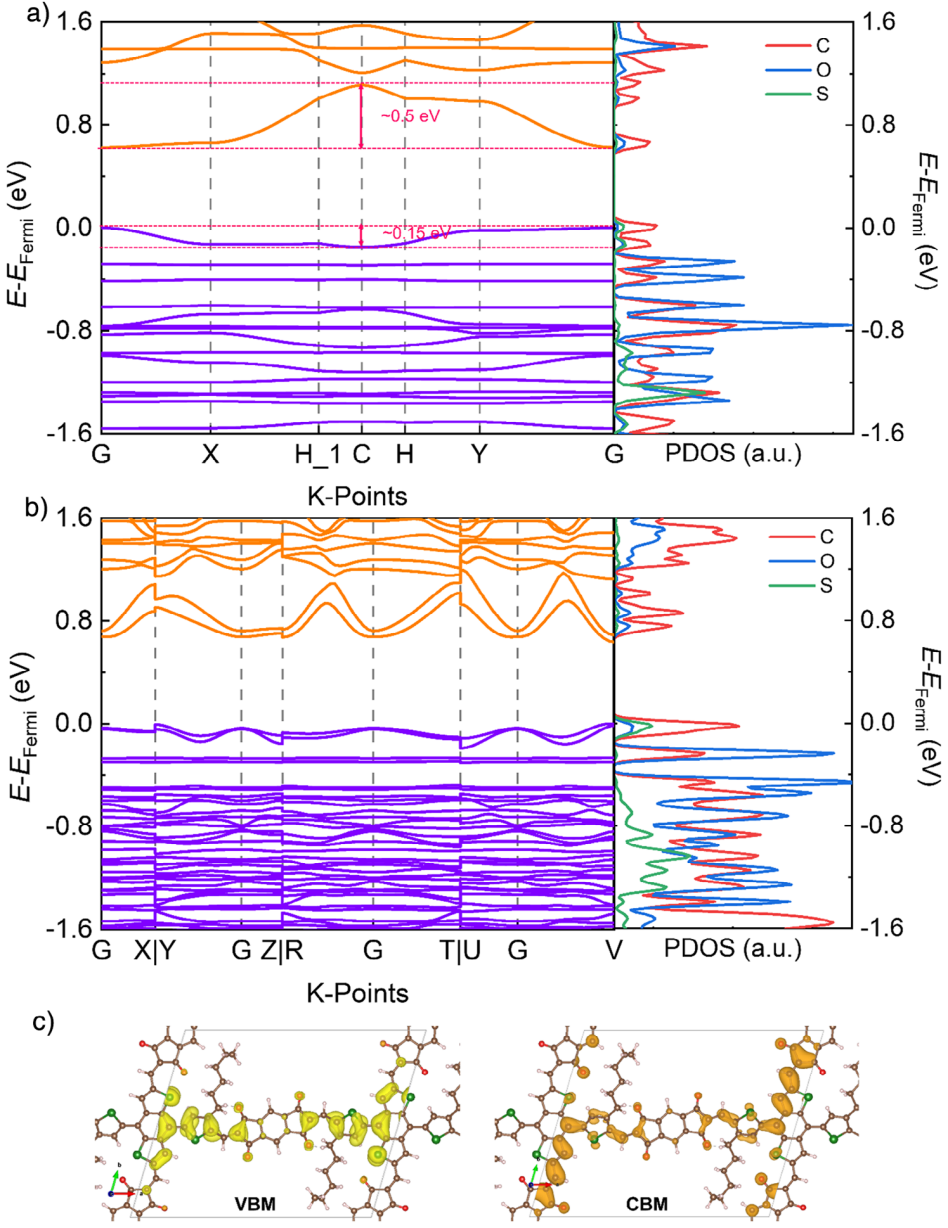
Electronic band structures. a,b) Band structures of monolayer and multilayer **2DPAV‐TBDT‐IT**, respectively. c) The partial charge density for monolayer **2DPAV‐TBDT‐IT**.

In order to investigate the conjugation efficiency, we measured the diffuse reflectance spectra of two 2D PAVs using UV–vis‐near IR spectroscopy.^[^ ^39^ ^]^ **2DPAV‐TBDT‐BT** presents a broad optical absorption in the range of ca. 400‒800 nm (Figure 4a), which corresponds to an optical band gap of 1.60 eV, as determined by the Tauc plot (Figure S21). By contrast, **2DPAV‐TBDT‐IT** exhibits an intense absorption band beyond 900 nm, corresponding to a much narrower band gap of 1.15 eV (Figure 4a and Figure S22). This observation can be attributed to the intramolecular charge transfer between the TBDT and IT units. The achieved band‐gap value of 1.15 eV is significantly lower than the reported 2D c‐COFs (e.g., ∼1.6 eV for the reported 2D PAVs).^[^ ^40^ ^]^

**Figure 4:**
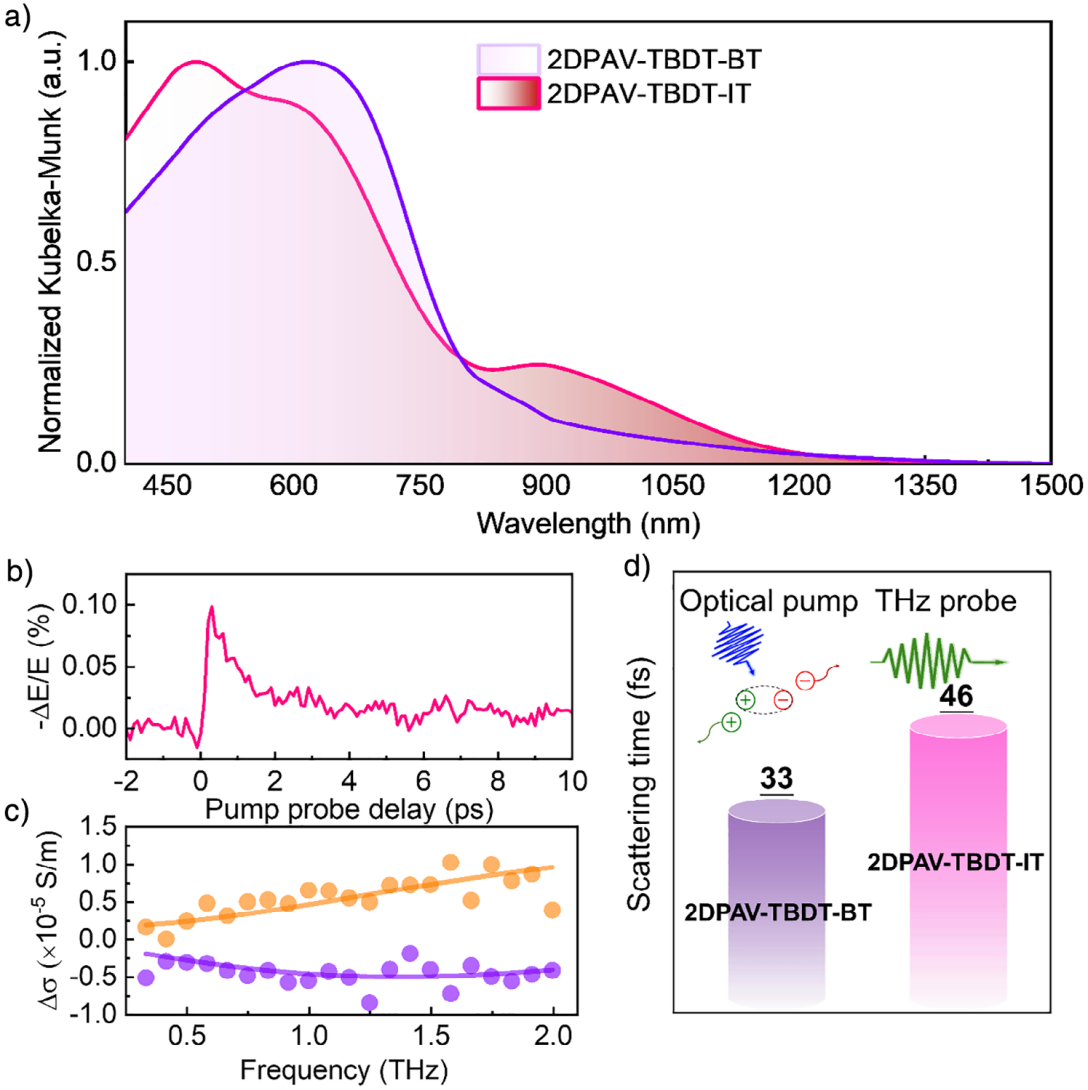
Charge transport properties of **2DPAV‐TBDT‐IT**. a) Diffuse reflectance spectra. b,c) Time‐ and frequency‐resolved photoconductivity, respectively. d) Comparison of scattering time between **2DPAV‐TBDT‐BT** and **2DPAV‐TBDT‐IT**.

Ultrafast THz spectroscopy is then applied to investigate the intrinsic charge transport properties. Following optical excitation, the photoconductivity dynamics of **2DPAV‐TBDT‐IT** exhibit a rapid rise due to the free carrier injection by the above‐band gap excitation under 400 nm pulsed laser followed by a quick decay within 2 ps (Figure 4b), presumably due to charge localization and/or electron‐hole recombination. The frequency‐resolved THz photoconductivity was also measured, which can be well‐described by the Drude–Smith model (Figure 4c). The fitting yields a confinement parameter (*c*) of −0.938 and a scattering time (*τ*) of 46.5 fs for the charge carriers. Taking together the effective mass, *τ*, and *c*, we estimate excellent charge mobility of 26 cm^2^ V^−1^ s^−1^ for the powder sample of **2DPAV‐TBDT‐IT**. Compared to **2DPAV‐TBDT‐BT** (with a *τ* of 33 fs, Figure 4d), the extended *τ* of **2DPAV‐TBDT‐IT** indicates a better transport ability, which might be induced by the well‐defined donor–acceptor carrier transporting channels.^[^ ^41^ ^]^

SO_2_ is a hazardous gas that poses significant risks to human health and the environment. However, achieving effective SO_2_ detection at ambient conditions remains a considerable challenge due to the insensitivity of SO_2_ to conventional sensor materials, which is ascribed to insufficient charge transfer between analyte and sensor material.^[^ ^42^ ^]^ The strong electron affinity of **2DPAV‐TBDT‐IT** with low‐lying LUMO localized on the IT unit (Figure 3c) can ensure a facile electron injection from SO_2_ to the 2D PAV, thereby boosting its sensitivity toward SO_2_. Meanwhile, the porous nature of the material can facilitate mass transport during sensing, and its high charge mobility can enable a fast charge transfer for signal transduction. Given these advantages and the electron‐dominating transport, we envisioned that **2DPAV‐TBDT‐IT** should be a promising semiconducting material for SO_2_ detection.

We thus fabricated the chemiresistors by drop‐casting **2DPAV‐TBDT‐IT** dispersion onto the interdigitated electrode area of pre‐patterned chips with a channel width and gap of 4 and 3 µm, respectively^[^ ^43^ ^]^

(Figure 5a). The sensing response to SO_2_ gas was studied using air or nitrogen as carrier gas (see details in Supporting Information). Upon exposure to SO_2_, the response of the sensor increased sharply, which corresponds to a decreased resistance of the sensor material (Figures S26–S29), indicative of partial electron transfer from SO_2_ to **2DPAV‐TBDT‐IT** (see more detailed discussion on sensing mechanism in Figures S23–S25). Upon exposure to 10 ppm SO_2_ at 100 °C for 50 s in air, the sensor's response reached as high as 96%, followed by an exceptional recovery process within 50 s (Figure 5b and Figure S28). Both at 60 and 80 °C, the sensors showed similar sensing behavior to that at 100 °C (Figure 5b, see the data for 120 °C in Figures S29 and S31). It is noteworthy that, at room temperature, **2DPAV‐TBDT‐IT** still exhibited a prominent response to SO_2_ with an intensity of 43% after an exposure of 50 s, while its recovery remained as good as that at high temperatures (Figure 5c, Figure S32). Based on the concentration‐dependent responses obtained under the best conditions, we can infer the sensor's limit of detection (LOD) using the root‐mean‐square deviation (inset of Figure 5d). The fitting of the results provides an impressive LOD of 0.088 ppb for **2DPAV‐TBDT‐IT** (Figure 5d), significantly surpassing the reported chemiresistive SO_2_ sensors (Table S2; 2D c‐COFs, MOFs, and MOF composites; LOD: 5–625 000 ppb).^[^ ^44^, ^45^, ^46^ ^]^ This result highlights the great potential of electron‐deficient 2D PAVs in ultrasensitive chemiresistors.

**Figure 5:**
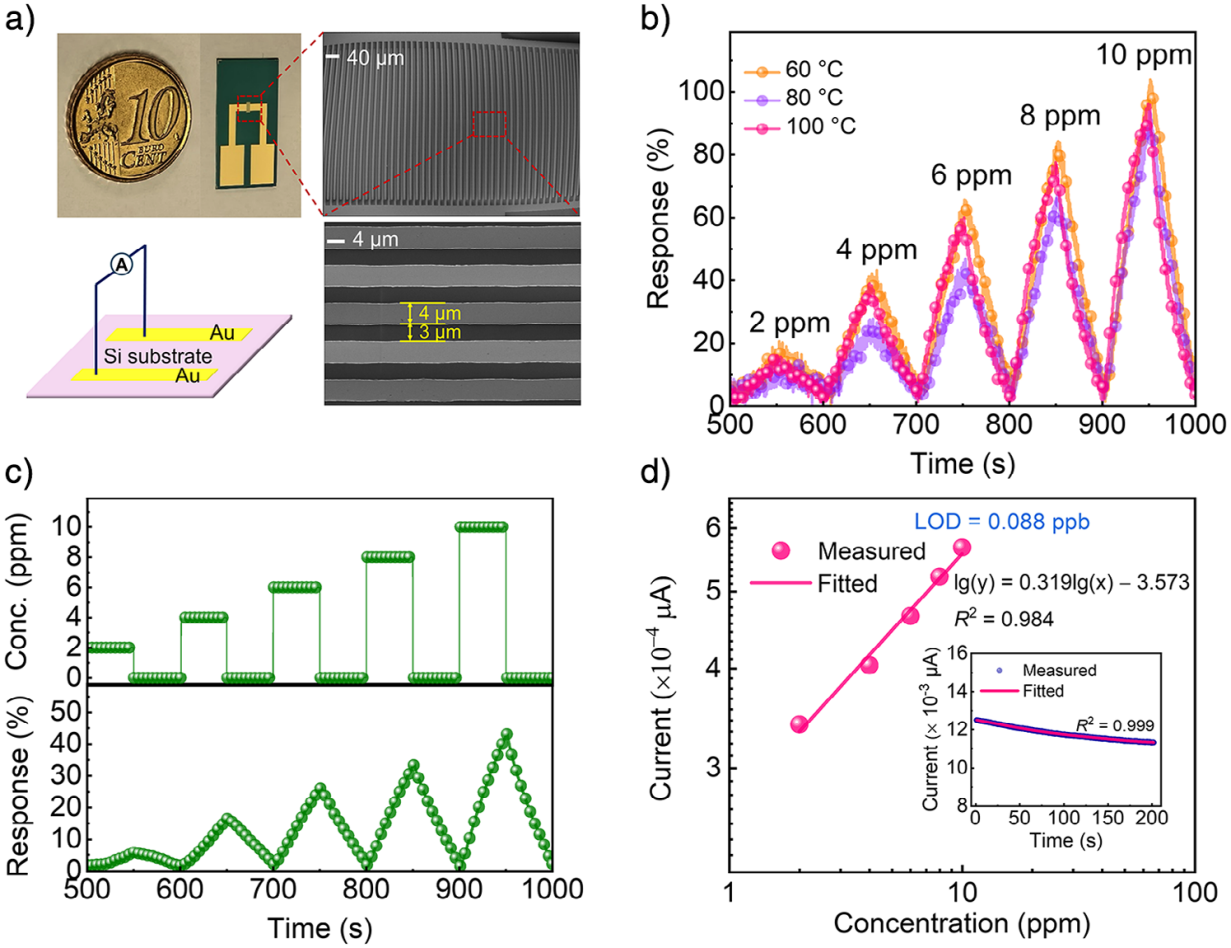
Chemiresistors based on **2DPAV‐TBDT‐IT** for ultrasensitive SO_2_ detection in air. a) Optical and SEM images of the fabricated chemiresistor. b,c) Concentration‐dependent response and recovery curves at high temperatures and room temperature, respectively. d) The linear relationship between response and SO_2_ concentration at 100 °C.

In summary, we have demonstrated the efficient synthesis of the novel TBDT/IT‐based donor–acceptor‐type 2D PAV. The achieved material exhibits effective conjugation associated with strong electronic band dispersion, leading to an optical band gap narrower than the reported 2D c‐COFs. Moreover, theoretical calculations and THz spectroscopy indicate a unique electron‐dominating transport for the 2D PAV with a high charge mobility. Using it as a sensor material, the **2DPAV‐TBDT‐IT** shows a high sensitivity to SO_2_ owing to facile electron injection from SO_2_ to the LUMO of the material. The fabricated chemiresistors exhibit fast response/recovery for SO_2_ detection at room temperature with an ultralow LOD. Further structural designs incorporating strong electron acceptors into 2D PAVs will be explored to enhance the 2D conjugation, charge carrier separation efficiency, and charge mobility for future electronics and optoelectronics.

## Conflict of Interests

The authors declare no conflict of interest.

## Supporting information



Supporting Information

## Data Availability

The data that support the findings of this study are available from the corresponding author upon reasonable request.
