# Supplementary material for: A Donor–Acceptor‐Type Two‐Dimensional Poly(Arylene Vinylene) for Efficient Electron Transport and Sensitive Chemiresistors
Source: Angew Chem Int Ed Engl. 2025 May 2;64(24):e202504302. doi: 10.1002/anie.202504302 (PMC12144878; doi:10.1002/anie.202504302)
Supplement: Supplementary file 1 — Supporting Information [file ANIE-64-e202504302-s001.pdf]

Supporting Information

A Donor-Acceptor-Type Two-Dimensional Poly(arylene vinylene) for Efficient Electron Transport and Sensitive Chemiresistors

Ruyan Zhao,‡ Wei Wang,‡ Yamei Liu,‡ Petko Petkov, Arafat Hossain Khan, Lei Gao, Peng Zhang, Eike Brunner, Hai I. Wang, Shivam Singh, Shirong Huang, Luis Antonio Panes-Ruiz, Yana Vaynzof, Mischa Bonn, Gianaurelio Cuniberti,* Mingchao Wang,* and Xinliang Feng*

Table of Contents

Section A. Methods

A1. Instrumentation

A2. Experimental Details

A3. DFT Calculation Details

Section B. Materials and Synthetic Procedures

Section C. Supporting Figures

Section D. Supporting Tables

Section E. References

Summary of Supporting Figures and Tables

Figure S1. The optimized molecular configurations of **M1−M7**

Figure S2. The optimized Kohn-Sham molecular orbitals of **M1−M7**

Figure S3. SEM image of **2DPAV-TBDT-IT**

Figure S4. FT-IR of m-TBDT, IT and **2DPAV-TBDT-IT**

Figure S5. ss-CPMAS ^{1}H NMR spectrum of **2DPAV-TBDT-IT**

Figure S6. pXRD of **2DPAV-TBDT-BT**

Figure S7. SEM image of **2DPAV-TBDT-BT**

Figure S8. FT-IR of m-TBDT, BT and **2DPAV-TBDT-BT**

Figure S9. Thermogravimetric analysis of **2DPAV-TBDT-IT**

Figure S10. pXRD patterns of **2DPAV-TBDT-IT** before and after drying at 100 ℃ overnight

Figure S11. pXRD patterns of **2DPAV-TBDT-IT** after soaking in different solvents

Figure S12. pXRD patterns of **2DPAV-TBDT-BT** after soaking in different solvents

Figure S13. FT-IR spectra of **2DPAV-TBDT-IT** after soaking in different solvents

Figure S14. FT-IR spectra of **2DPAV-TBDT-BT** after soaking in different solvents

Figure S15. BET plots of **2DPAV-TBDT-IT**

Figure S16. Wall-to-wall distances in **2DPAV-TBDT-IT** model

Figure S17. BET plots of **2DPAV-TBDT-IT** after stability treatment

Figure S18. BET pplots of **2DPAV-TBDT-BT**

Figure S19. EDS mapping pictures of **2DPAV-TBDT-IT**

Figure S20. Band structure of **2DPAV-TBDT-BT**

Figure S21. Diffuse reflectance spectrum and the Tauc plot of **2DPAV-TBDT-BT**

Figure S22. Diffuse reflectance spectrum and the Tauc plot of **2DPAV-TBDT-IT**

Figure S23. Response-recovery curves of **2DPAV-TBDT-IT** under air and nitrogen conditions

Figure S24. Adsorption field of SO_2 for **2DPAV-TBDT-IT**

Figure S25. XPS spectra before and after SO_2 sensing cycles

Figure S26. Response-recovery curve of **2DPAV-TBDT-IT** at 60 °C

Figure S27. Response-recovery curve of **2DPAV-TBDT-IT** at 80 °C

Figure S28. Response-recovery curves of **2DPAV-TBDT-IT** at 100 °C

Figure S29. Response-recovery curves of **2DPAV-TBDT-IT** at 120 °C

Figure S30. Representative response-recovery curves of **2DPAV-TBDT-IT** at 100 °C
Figure S31. Representative response-recovery curves of **2DPAV-TBDT-IT** at 120 °C
Figure S32. Representative response-recovery curves of **2DPAV-TBDT-IT** at RT
Figure S33. Response-recovery curves of **2DPAV-TBDT-IT** at different temperatures
Figure S34. The highest response (%) of **2DPAV-TBDT-IT** at different temperatures
Figure S35. Raman spectra before and after SO_2 sensing cycles
Figure S36. ^{1}H NMR spectrum of **TBDT**
Figure S37. ^{1}H NMR spectrum of **m-TBDT**
Figure S38. ^{13}C NMR spectrum of **m-TBDT**
Figure S39. ^{13}C-CP MAS NMR spectra of **m-TBDT** and **2DPAV-TBDT-IT**
Table S1. Starting materials and resources
Table S2. Condition screening for the synthesis of crystalline **2DPAV-TBDT-IT**
Table S3. Summary of sensing performance

Section A. Methods

A1. Instrumentation

Nuclear magnetic resonance (NMR) spectroscopy. Solution-state ^{1}H NMR spectra were measured on a BRUKER AVANCE III 300 MHz spectrometer. Solid-state MAS NMR spectra were acquired by a Bruker Advance 800 MHz spectrometer and a commercially available 1.3 mm double-resonance magic angle spinning (MAS) probe at 50 kHz. ^{1}H Depth MAS spectra were recorded at 2 µs pulse length and 3 s delay time. The probe background signal is suppressed by using a Depth pulse sequence.[1] For the ^{13}C spectrum, 60 K scans are collected at a delay time of 3 s (more than five times of ^{1}H-T_1) and 3 ms and 50 µs contact time with a ramped ^{1}H decoupled (SPINAL-64) cross-polarization (CP) pulse sequence at a resonance frequency of 201.23 MHz. Short contact time ^{13}C-NMR spectra suppressed signal from quaternary and mobile aliphatic ^{13}C resonance, thereby assisting in the assignment of the spectrum. ^{13}C spectra are simulated by using ACD/Labs software. All spectra were referenced to tetramethylsilane (TMS) used as a secondary reference for ^{13}C, resonating at 29.5 ppm.

Powder X-ray diffraction (pXRD) analysis. pXRD patterns were obtained on STOE STADI P diffractometer with Cu Kα line focused radiation at 40 kV and 40 mA from 2θ in the range of 2° to 40° with 0.02° increment.

Fourier-transform infrared (FT-IR) spectroscopy. FT-IR spectra were collected on a Bruker Tensor II spectrometer with a universal Zn-Se ATR (attenuated total reflection) accessory ranging from 400 cm^{-1} to 4000 cm^{-1}.

Thermogravimetric analysis (TGA). TGA from 20-1000 °C was carried out on a TG50 analyzer (Mettler-Toledo) in a nitrogen atmosphere using a 10 °C/min ramp without equilibration delay.

Scanning electron microscopy (SEM). SEM (FESEM, Zeiss Gemini 500) was used to investigate the structure of the as-synthesized 2D PAVs at an accelerating voltage of 3.0 and 3.5 kV. Powder samples were dispersed in ethanol and then dropped onto silicon substrates, which were attached with conductive adhesive tapes to a flat aluminum sample holder and then coated with gold.

Nitrogen physisorption measurements. N_2 physisorption isotherms were recorded by a Micrometrics ASAP 2020-M surface area analyzer up to 1 bar at 77 K. Before measurements,

samples were activated for 12 h at 120 °C. The pore size distribution was analyzed using the nonlocal density functional theory (NLDFT) method.

A2. Experimental Details

Chemical stability test. The samples were dispersed in different solvents, such as methanol, DMF, HCl (37%, 12 M), and KOH (12 M) for 24 hours. Before FT-IR spectra and pXRD measurement, precipitates were collected by filtration, washed with anhydrous acetone, and dried at 120 °C under vacuum overnight.

Preparation of gas sensors. The prepared material was dispersed in 2 mL solvent consisting of 1 ml of DI water and 1 ml of ethanol for 2 mg each, respectively, and sonicated for 10 min to make it uniformly dispersed in solvent. Then a certain amount of suspension was extracted and deposited between gold electrodes on a Si substrate. Finally, the device was placed on a hot plate at 40 °C for 3 h to remove the solvent. The prepared sensor device was placed in a vacuum oven, activated at 100 °C for 4 hours, cooled to room temperature, and stored in a drying cabinet for testing.

Photoconductivity measurements by THz Spectroscopy. In a typical optical pump-THz probe (OPTP) experiment, an optical pulse (‘pump’, 255 μJ cm^{-2} at 400 nm) was firstly used to photoinject electrons and holes in the conduction and valence bands, respectively. Subsequently, a ~1 ps THz pulse (‘probe’) with electric field $E_0(t)$ was sent collinearly through the sample at a pump-probe delay time t_p, leading to the attenuation of THz electric field ($-\Delta E(t)$). The relative attenuation is proportional to the photoconductivity $\Delta\sigma$ of the sample, i.e.,

$$-\frac{\Delta E}{E} \propto \Delta\sigma = \mathrm{ne\mu} \quad (1)$$

(n is the photoexcited carrier density, e is the elementary charge, μ is the charge mobility). Fourier transform can further obtain the complex frequency-resolved photoconductivity spectrum $\Delta\sigma(\omega)$. The photoconductivity spectra are all fitted by the modified Drude model, the so-called Drude-Smith model, which reads:

$$\sigma = \frac{\varepsilon_0\omega_p^2\tau}{1-i\omega\tau}\left(1 + \frac{c}{1-i\omega\tau}\right) \quad (2)$$

Here, ε_0 is the static dielectric constant, ω angular frequency, ωp plasma frequency, τ Drude-Smith scattering time, c back scattering parameter ($-1 \leq c \leq 0$). c is introduced to account for the confinement effect to the charge transport from e.g., grain boundaries.With $c = -1$, the charges are

subject to the complete back scattering; While $c = 0$, the charges will scatter isotropically, returning to the classical Drude model. This model has been widely applied to describe the charge transport in COFs, metal-organic frameworks (MOFs), and other organic or inorganic materials.11-13 THz spectroscopy measures the microscopic charge transport within the cycle of the THz pulse (~1 ps), corresponding to a transport length of tens of nm. This indicates that we measure the local charge carrier mobility inside the crystalline domains, where the motion of these charges is mainly affected by e.g., defects and phonons.

Gas sensing measurement. SO_2 used in this work was purchased from an air products company (UK) and was all pre-diluted in nitrogen under absolutely dry conditions. The compressed air with class 0 (oil-free) was used as the balance gas to create a comparative experimental group. During the experiments, the desired concentrations of analytes were precisely prepared by adjusting the analyte-to-air ratio using a gas mixing system (MCQ Instruments GB-103). The constant bias voltage on the sensor was 6 V and the electrical signal was recorded by a Keithley 2450 source-meter in real-time. A 1 cm × 1 cm heated ceramic plate was used to provide the sensor with different working temperatures. The responses (R(%)) of all samples to the analytes were determined as detecting the relative change in resistance, which is defined as:

$$R(\%) = (\frac{R(0)}{R(t)} - 1) * 100 \quad (3)$$

(where R(t) is the real-time resistance of the sample in analytes diluted in air, and R(0) is the initial resistance in the air before testing). The response and recovery times for the sample were acquired as the times taken to achieve 90% of the total resistance change.

Signal noise characterization and LOD calculation. The noise of developed sensors was deduced from the root-mean-square deviation (RMS) at the baseline following 5th-order polynomial fitting. According to the IUPAC definition[2], when the signal-to-noise ratio equals 3, the signal is considered to be a true signal. Therefore, the detection limit can be extrapolated from the linear calibration curve when the signal equals 3 times the noise[3]. Linear regression provided an equation of best-fit (slope = s). With these values, we extrapolated the theoretical LOD from (4):

$$LOD = 3 * \frac{RMS}{s} \quad (4)$$

A3. DFTB and DFT Calculation Details.

Since the structure of the 2D polymer is relatively large, we applied the self-consistent-charge density-functional tight-binding (SCC-DFTB) approach to find the most suitable atomistic monolayer and multilayer models. For this purpose, DFTB+ software was used with a 3ob-3-1 parameter set[4]. At first, a monolayer model of **2DPAV-TBDT-IT** was constructed from scratch. After the monolayer model was fully optimized, various multilayer models were created in AA-eclipsed, serrated, AB, and ABC stacking modes. All models were fully optimized (cell and lattice vectors) at the SCC-DFTB level. After a detailed comparison of all models' experimental XRD patterns and simulated XRD patterns, the most appropriate multilayered model was selected for further analysis.

The electronic properties (band structure and density of states) of monolayer and multilayer models were calculated with the Density functional theory (DFT) using the Vienna ab initio Simulation Package (VASP) version 5.4.1[5,6]. The electronic wave functions were expanded in a plane-wave basis set with a kinetic energy cutoff of 500 eV. Electron-ion interactions were described using the projector augmented wave (PAW) method[7,8]. Generalized gradient approximation (GGA) of the exchange-correlation energy in the form of Perdew-Burke-Ernzerhof (PBE)[9] was applied, and range-separated hybrid exchange-correlation functional of HSE06 form[10]. A Monkhorst-Pack Gamma-centered grid[11] with 2×2×1 dimension was used for K-point sampling of the Brillouin zone for the monolayer. In the computational protocol for the 3D stacking, the K-point grid dimension was changed to 2×3×1, and Grimme-D2 correction for the dispersion interlayer interactions was applied[12]. The 2D PAV monolayer was modeled by adding an ample vacuum space, 10 Å, in the direction normal to the monolayer. The unit cell used in the calculations of the 3D models contains two layers.

We used ORCA 5.0 with the hybrid exchange-correlation functional B3LYP[13–16] to study an isolated cluster model. The orbital basis sets of def2-TZVP[17] form with auxiliary basis sets AuxJ[18], AuxC[18], and AuxJK[18] were used.

Binding energies to the periodic structure of SO_2 were calculated using VASP code with Generalized gradient approximation of the exchange-correlation energy in the form of Perdew-Burke-Ernzerhof (PBE), including Grimme-D2 correction for the dispersion interactions. For

better accuracy, the electronic wave functions were expanded in a plane-wave basis set with a higher kinetic energy cutoff (600 eV).

Section B. Materials and Synthetic Procedures

General

Unless otherwise stated, all commercially available chemicals were used as received without further purification. The reactions were performed using standard vacuum-line and Schlenk techniques. Purification of all compounds was performed under air with reagent-grade solvents.

Synthesis

Synthesis of 4,8-di(thiophen-2-yl)benzo[1,2-b:4,5-b']dithiophene (TBDT). **TBDT** was synthesized using an adapted method according to the previous report. ^{1}H NMR (CDCl$_3$, 300 MHz, 25 ^{o}C) δ 7.63 (d, J = 5.7 Hz, 2H), 7.46 (d, J = 5.6 Hz, 2H), 7.32 (d, J = 1.5 Hz, 2H), 7.11 (d, J = 1.4 Hz, 2H), 2.72 (t, J = 7.7 Hz, 4H), 1.72 (p, J = 7.4 Hz, 4H), 1.46–1.31 (m, 12H), 0.97–0.87 (m, 6H).

Synthesis of 4,8-bis(5-formyl-4-hexylthiophen-2-yl)benzo[1,2-b:4,5-b']dithiophene-2,6-dicarbaldehyde (m-TBDT). **m-TBDT** was synthesized using an adapted method according to the previous report. ^{1}H NMR (CDCl$_3$, 300 MHz, 25 ^{o}C) δ 10.18 (s, 2H), 10.12 (s, 2H), 8.35 (s, 2H), 7.48 (s, 2H), 3.17–3.05 (m, 4H), 1.80 (t, J = 7.6 Hz, 4H), 1.47–1.34 (m, 12H), 0.95–0.88 (td, J = 7.1, 5.8, 3.5 Hz, 6H). ^{13}C NMR (CDCl3, 75 MHz, 25 ^{o}C) δ 184.32, 182.08, 153.32, 146.19, 145.29, 141.26, 139.33, 137.80, 132.69, 132.27, 127.13, 31.58, 31.53, 29.08, 28.71, 22.62, 14.09.

Synthesis of 2DPAV-TBDT-IT. A 5 mL high-pressure glass tube was charged with **m-TBDT** (30.00 mg, 0.047 mmol), **IT** (20.24 mg, 0.095 mmol), mesitylene (2.4 mL), and 6 M AcOH (24 μL). The tube was sonicated at room temperature for 3 min, degassed by three freeze-pump-thaw cycles, sealed under vacuum and heated at 120 °C for three days. After cooling to room temperature, the precipitate was filtrated, washed with dimethylformamide, acetone, water, tetrahydrofuran, and anhydrous acetone, respectively, then collected and dried under vacuum at 120 °C overnight to get dark powders in 91% yield.

Synthesis of 2DPAV-TBDT-BT. A 5 mL high-pressure glass tube was charged with **m-TBDT** (12.00 mg, 0.019 mmol), **BT** (9.24 mg, 0.038 mmol), dioxane (1 mL) and NH_4OAc (12 mg, 0.15mmol). The tube was sonicated at room temperature for 5 min, degassed by three freeze-pump-thaw cycles, sealed under vacuum and heated at 120 °C for three days. After cooling to room temperature, the precipitate was filtrated, washed with dimethylformamide, H_2O, methanol and

acetone, respectively, then collected and dried under vacuum at 100 °C overnight to get dark powders in 95% yield.

Section C. Supporting Figures

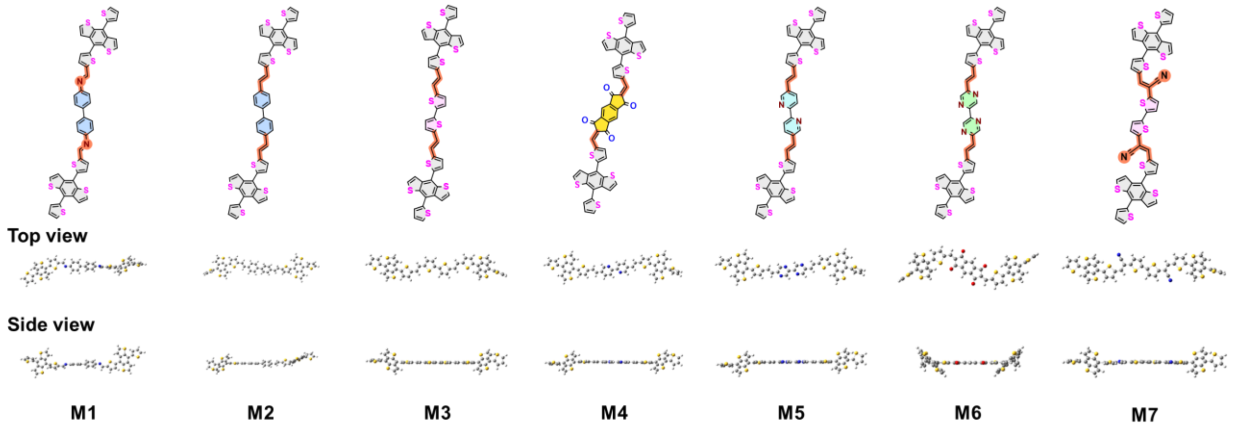

Figure S1. The optimized molecular configurations of **M1−M7**. Basis set: b3lyp-6-31g(d,p).

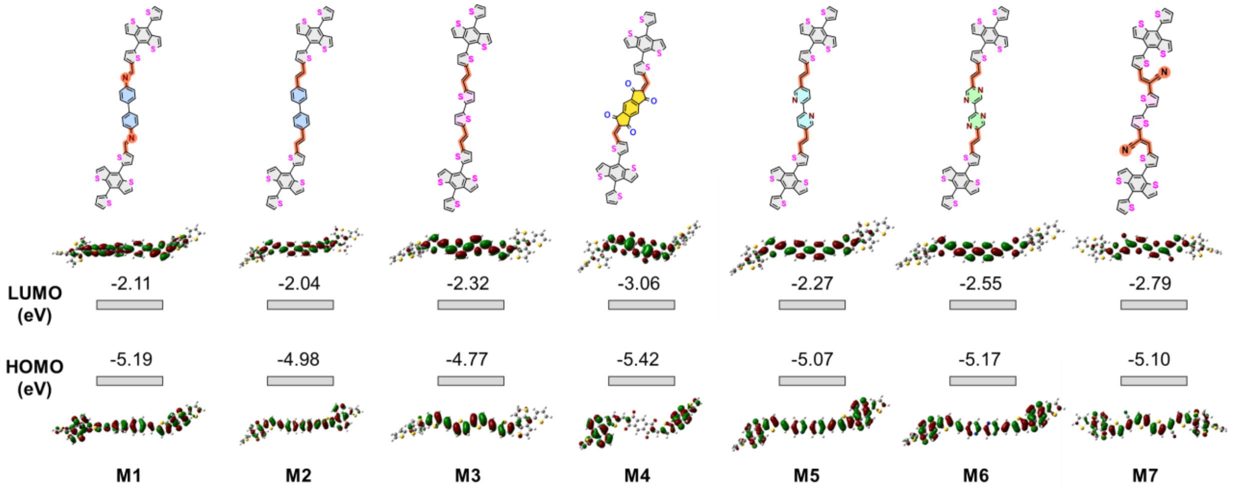

Figure S2. Chemical structures and the optimized Kohn-Sham molecular orbitals of **M1−M7**. Basis set: b3lyp-6-31g(d,p).

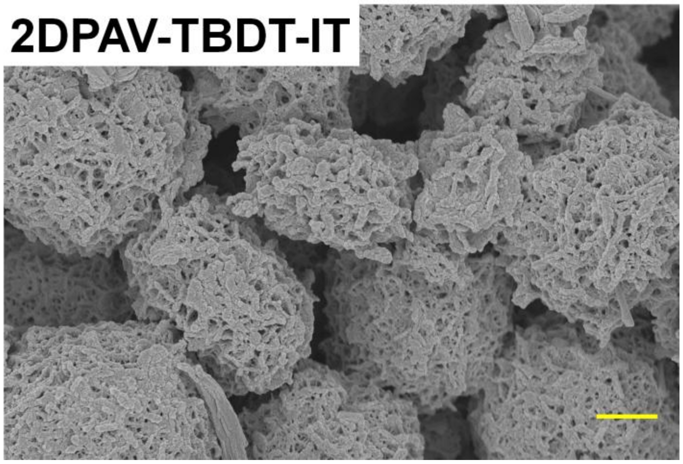

Figure S3. SEM image of pristine **2DPAV-TBDT-IT**. The scale bar represents 1 μm.

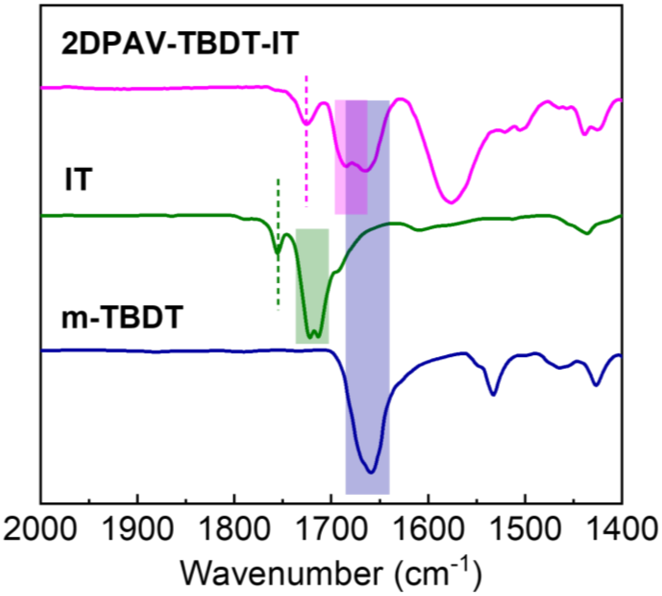

Figure S4. FTIR spectra of **2DPAV-TBDT-IT** (Zoom-in: 2000–1400 cm^{-1}**)**.

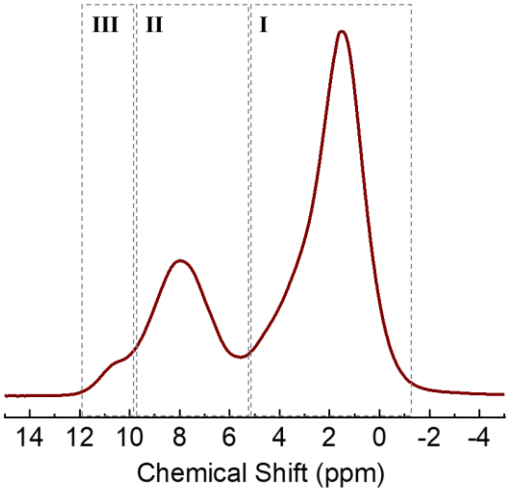

	Assignment	Total Area (%)
I	Aliphatic protons	74.01
II	Aromatic protons	23.75
III	remaining protons in the unreacted -CHO	2.24

Figure S5. ss-CPMAS-^{1}H NMR spectrum of **2DPAV-TBDT-IT**. The ratio of unreacted aldehyde groups is determined to be 2.24%.

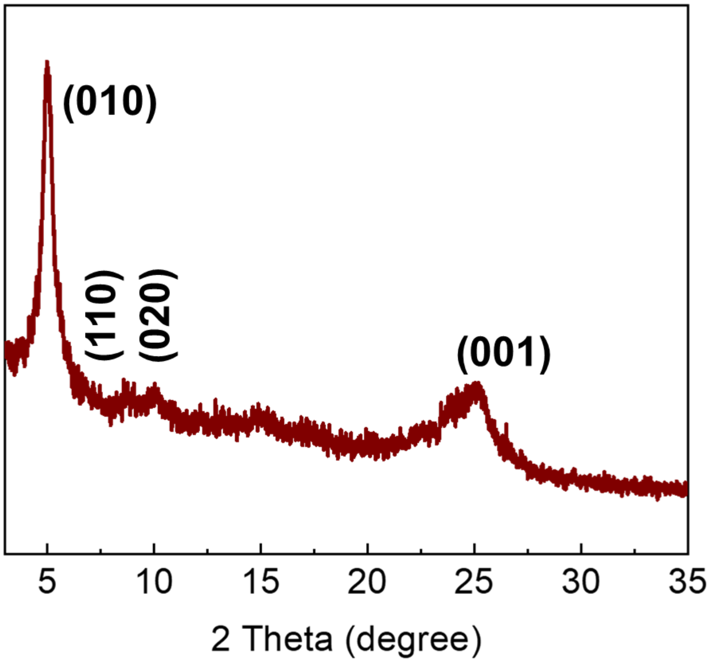

Figure S6. Measured pXRD pattern of **2DPAV-TBDT-BT**.

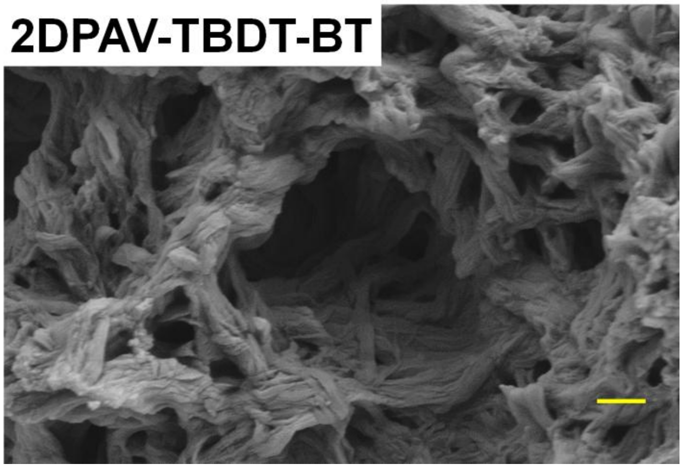

Figure S7. SEM image of pristine **2DPAV-TBDT-BT**. The scale bar represents 500 nm.

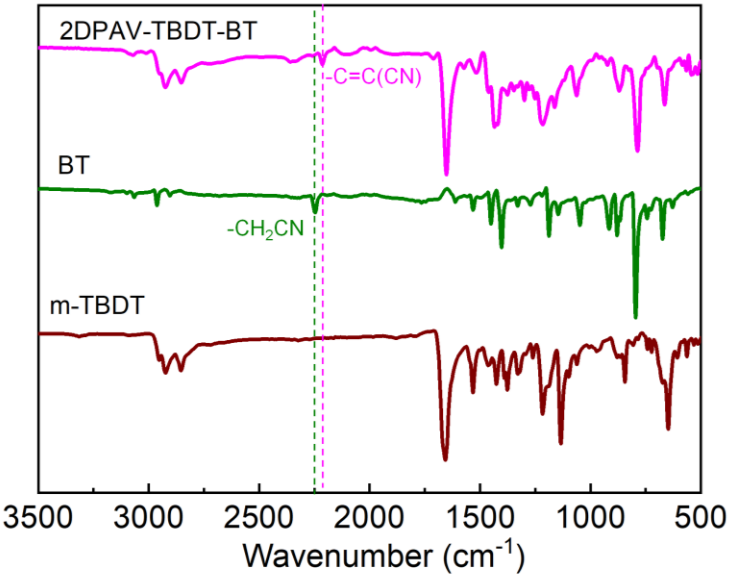

Figure S8. FT-IR spectra of **m-TBDT**, **BT**, and **2DPAV-TBDT-BT**.

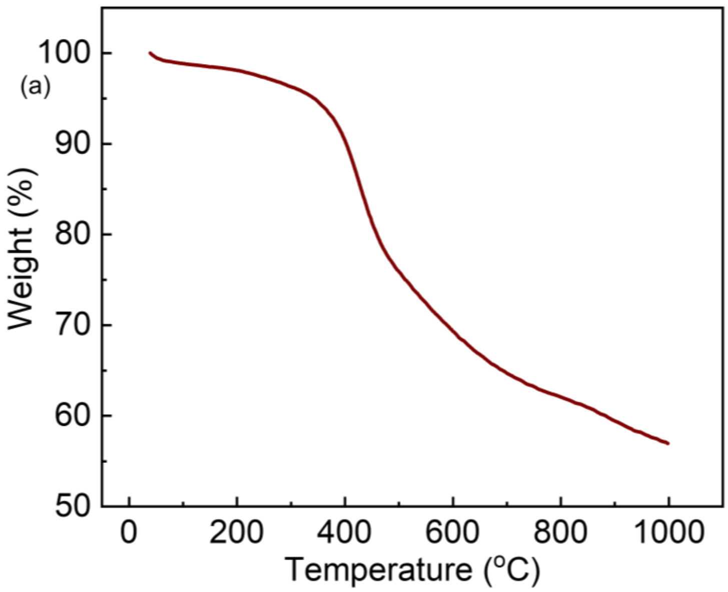

Figure S9. Thermogravimetric analysis of **2DPAV-TBDT-IT**.

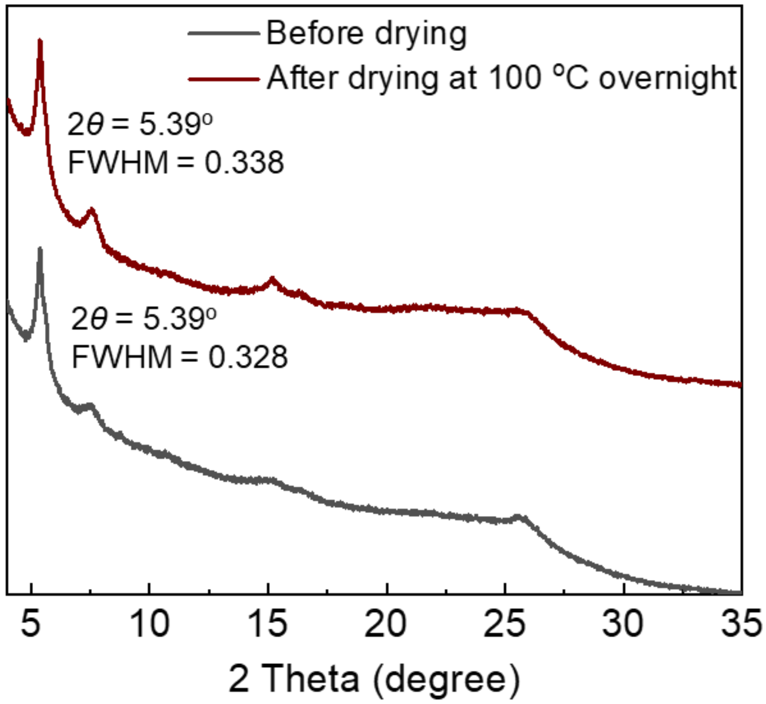

Figure S10. pXRD patterns of **2DPAV-TBDT-IT** before and after drying at 100 °C overnight.

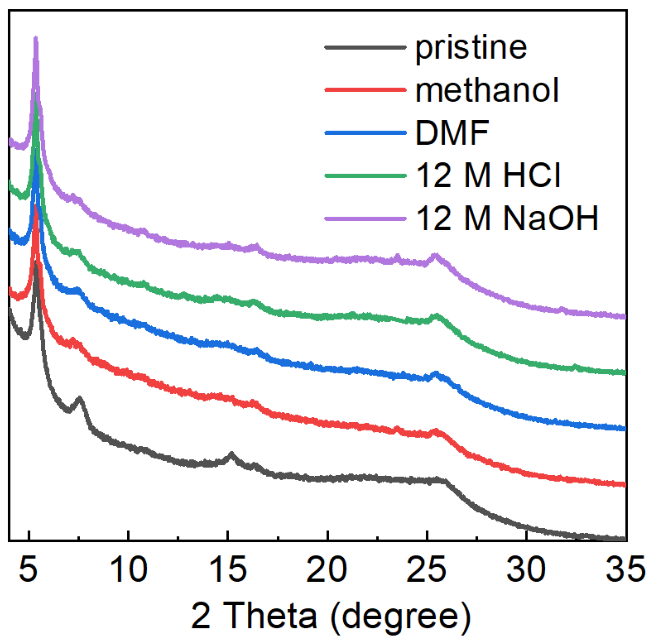

Figure S11. pXRD patterns of **2DPAV-TBDT-IT** after socking in different solvents.

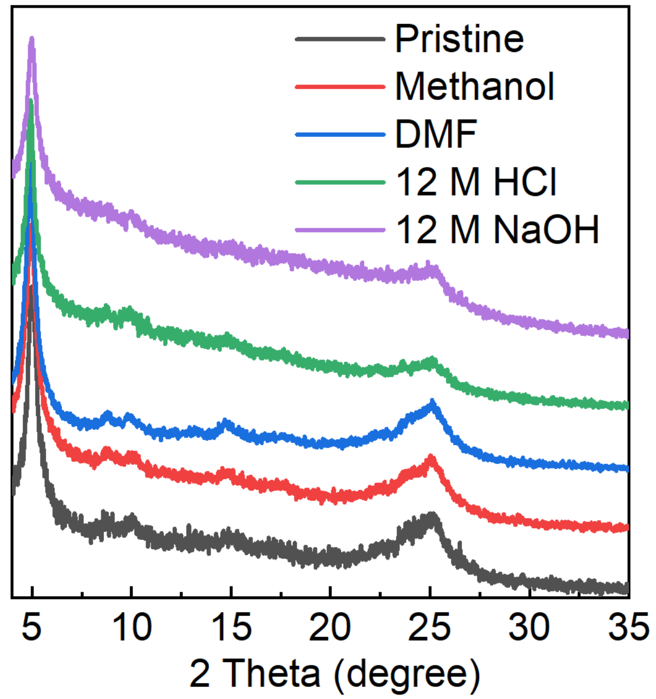

Figure S12. pXRD patterns of **2DPAV-TBDT-BT** after socking in different solvents.

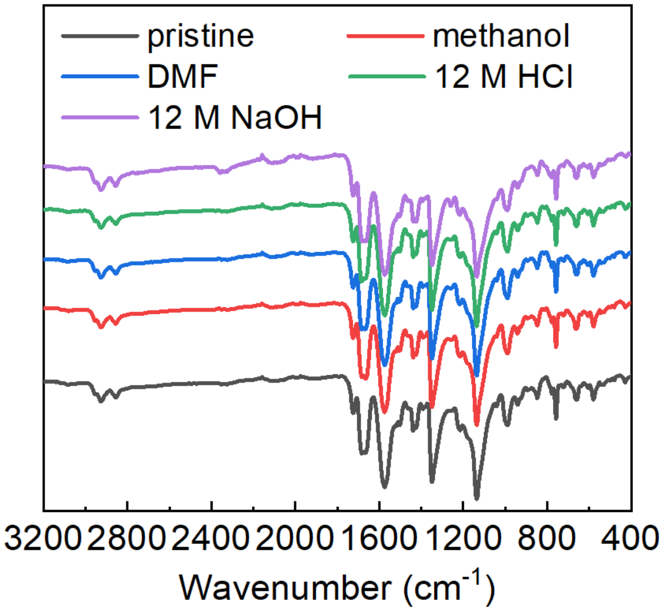

Figure S13. FTIR spectra of **2DPAV-TBDT-IT** after socking in different solvents.

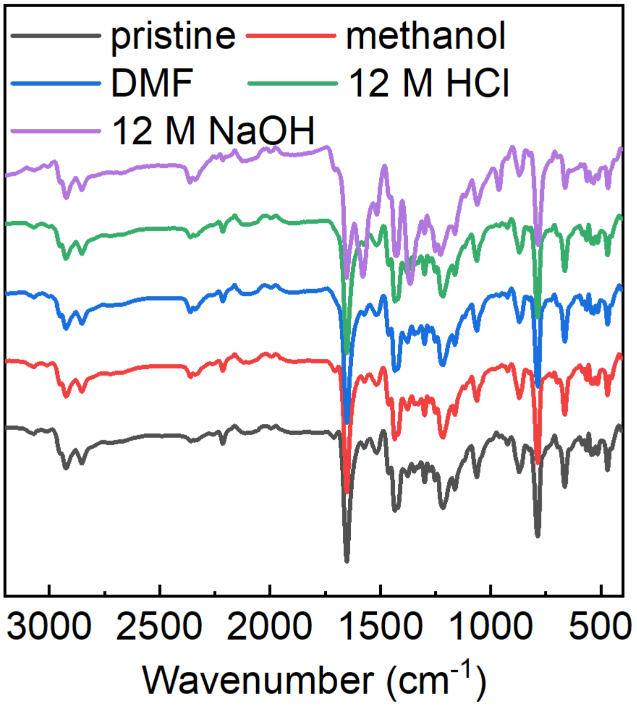

Figure S14. FTIR spectra of **2DPAV-TBDT-BT** after socking in different solvents.

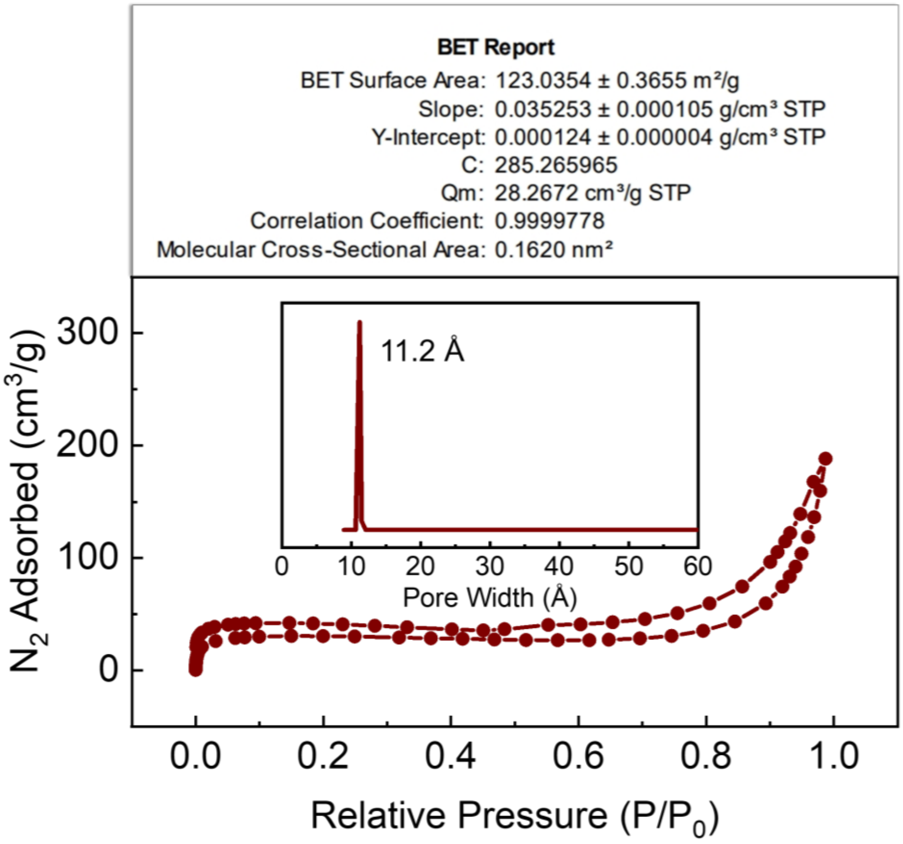

Figure S15. Nitrogen physisorption and pore size distribution of **2DPAV-TBDT-IT**.

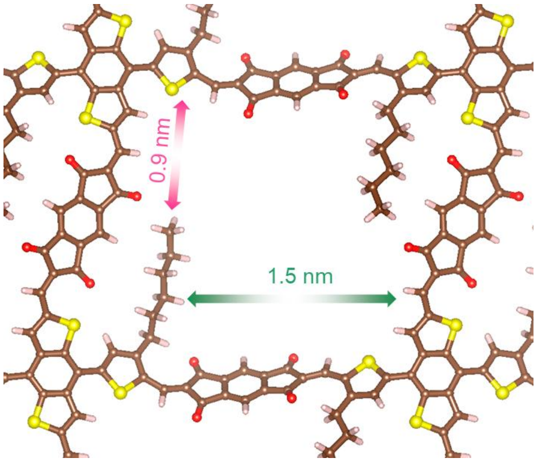

Figure S16. Depiction of wall-to-wall distances in **2DPAV-TBDT-IT** model.

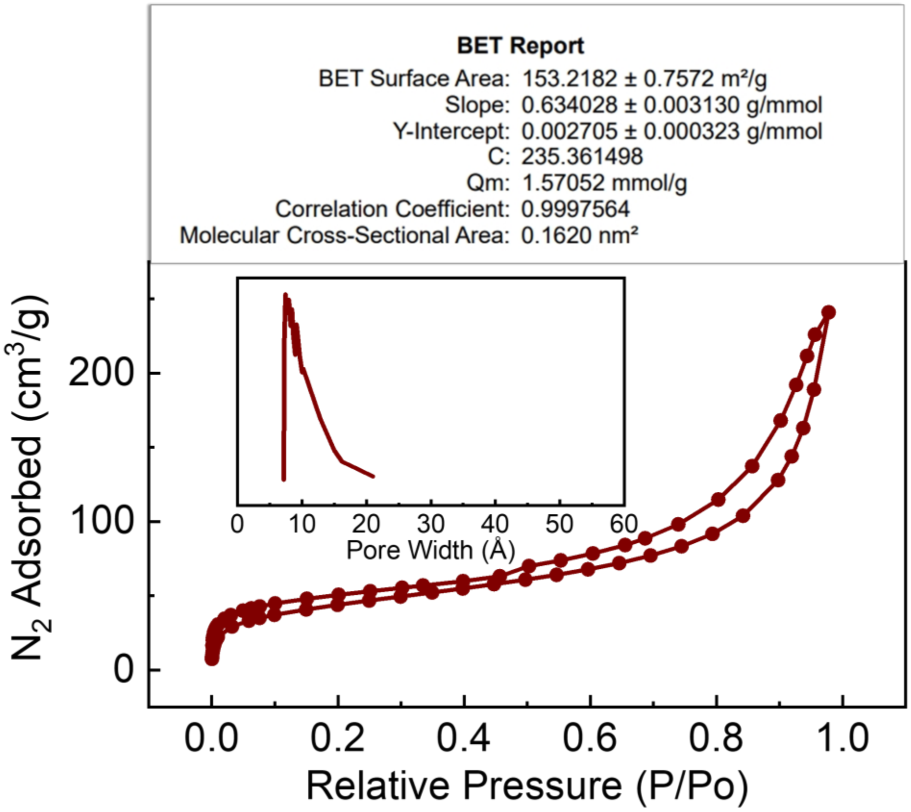

Figure S17. Nitrogen physisorption and pore size distribution of **2DPAV-TBDT-IT** after treatment with 12 M NaOH (aq.).

The Brunauer−Emmett−Teller (BET) surface area of **2DPAV-TBDT-IT** was estimated at about 123 m^2 g^{-1} (**Figure S15**). The C parameter is 285.27, and the correlation coefficient is 0.9999778. The main pore size was determined as 1.1 nm using the nonlocal DFT method, which falls between the smallest (0.9 nm) and the largest (1.5 nm) wall-to-wall distances of the framework (**Figure S16**). After NaOH (aq.) treatment, the BET surface area of **2DPAV-TBDT-IT** slightly increased from 123 m^2 g^{-1} to 153 m^2 g^{-1} (**Figure S17**).

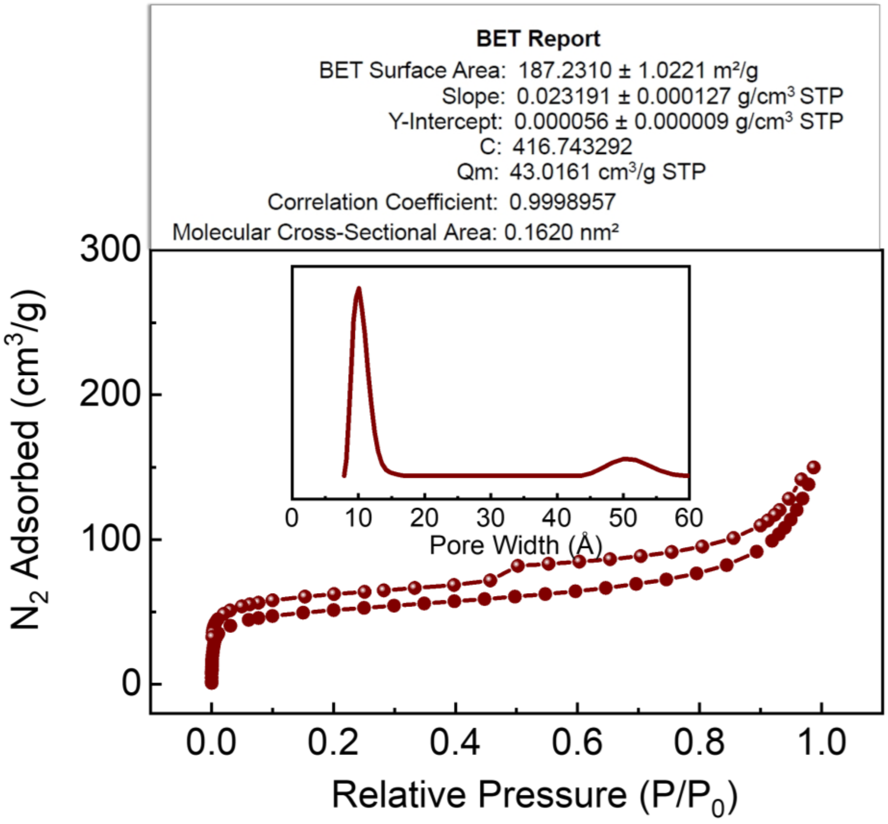

Figure S18. Nitrogen physisorption and pore size distribution of **2DPAV-TBDT-BT**[19].

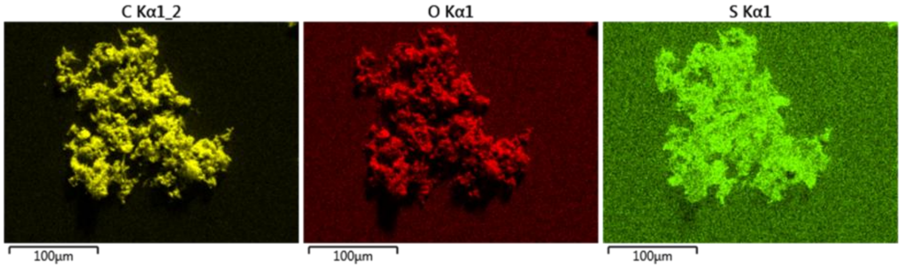

Figure S19. Energy dispersive X-ray spectroscopy (EDS) mapping pictures of **2DPAV-TBDT-IT**.

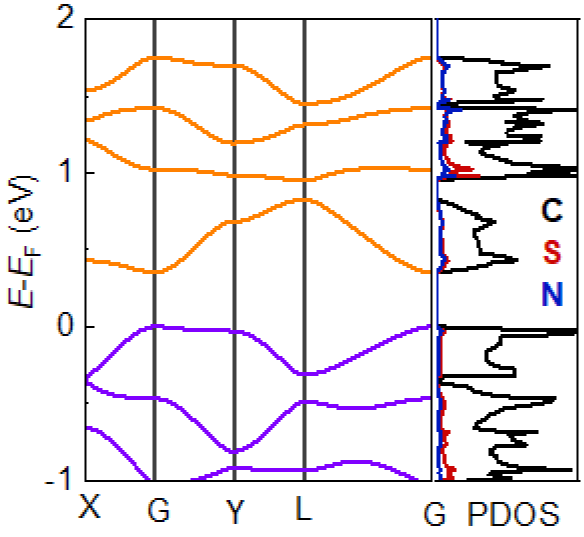

Figure S20. Electronic band structures and projected density of states (PDOS) of the multilayered model of **2DPAV-TBDT-BT**.[19]

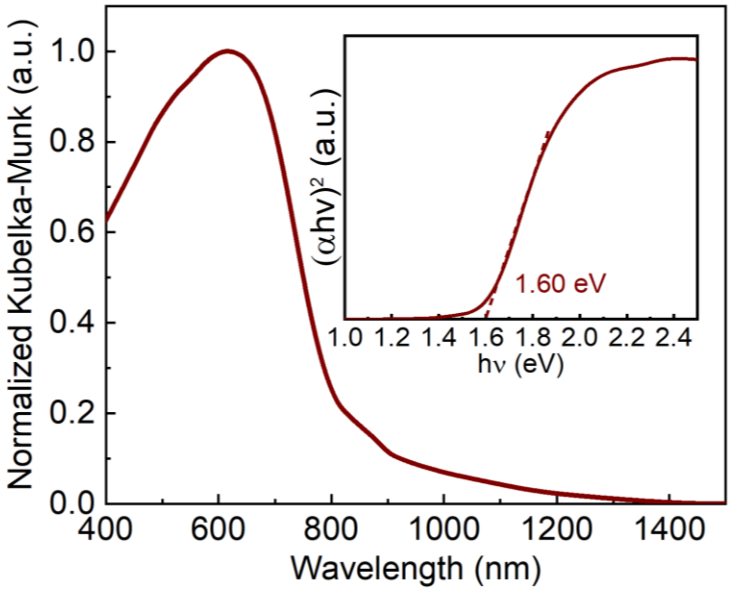

Figure S21. Diffuse reflectance spectrum and the Tauc plot of **2DPAV-TBDT-BT**.

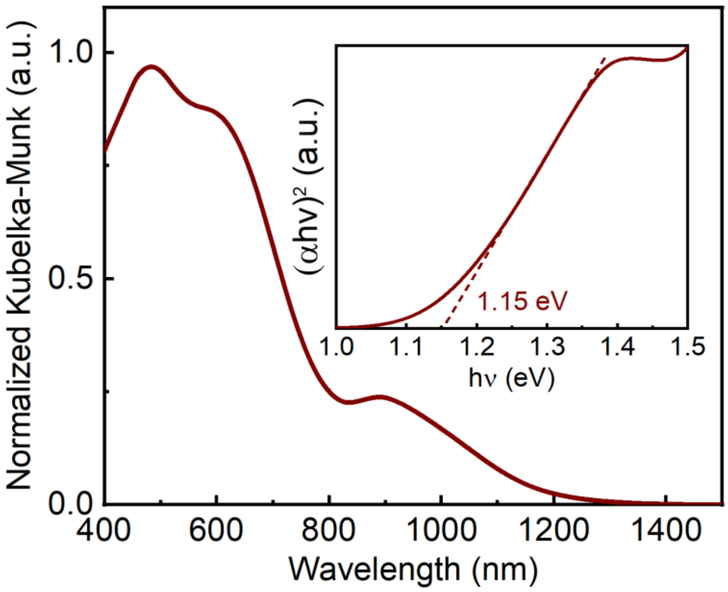

Figure S22. Diffuse reflectance spectrum and the Tauc-plot of **2DPAV-TBDT-IT**.

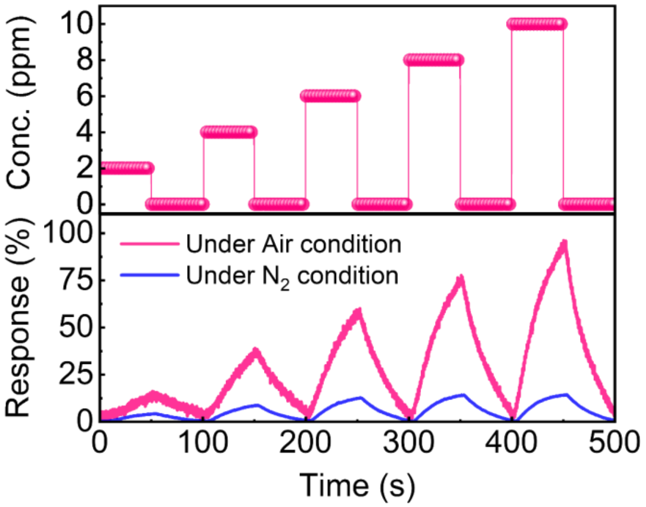

Figure S23. Response and recovery curves of **2DPAV-TBDT-IT** under air and nitrogen conditions at 100 °C upon various SO_2 concentrations (2, 4, 6, 8, and 10 ppm).

***Proposed mechanism*:**

As shown in Figure S20, the response measured in air is much higher than in nitrogen, suggesting oxygen plays a role in the sensing process. Electron depletion theory can be applied to interpret

the sensing mechanism. Oxygen molecules adsorbed on the sensor surface will extract electrons from the VBM of the **2DPAV-TBDT-IT** to form surface oxygen ions (O_2^-) (**Eq. 5**). When exposed to SO_2 gas, the reaction of SO_2 molecules and O_2^- releases electrons to the CBM of the **2DPAV-TBDT-IT** and generates SO_3 (**Eq. 6**), thereby reducing the thickness of the electron depletion layer and reducing the resistance.[20]

$$O_2\,(gas) + VBM + e^- \rightarrow O_2^-\,(ads.) \quad (5)$$

$$2SO_2\,(gas) + 2O_2^-\,(ads.) \rightarrow 2SO_3 + e^- + CBM \quad (6)$$

In this scenario, the porous nature of the **2DPAV-TBDT-IT** boosts the adsorption of gas molecules. Donor-acceptor molecular geometry generates a lot of intramolecular heterojunctions to facilitate electron-hole separation, and the strong electron-deficient IT unit further enhances the electron transfer from SO_2 to the surface of the **2DPAV-TBDT-IT**. The high charge mobility can enable a fast charge transfer for signal transduction. Thus, the sensors exhibit prominent sensing performance without building p-n heterojunctions using p- and n-type semiconductors.

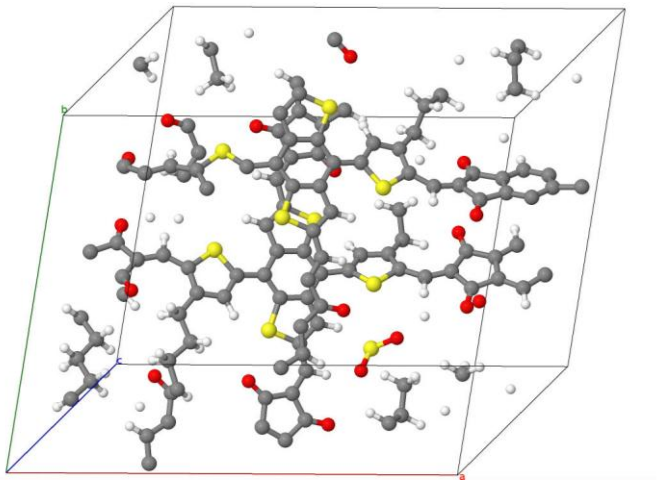

Figure S24. Adsorption field of SO_2 for **2DPAV-TBDT-IT**.[21]

To probe different possible locations of the SO_2 in the periodic models of 2DPAV-BDT-IT, various initial locations of SO_2 were used. The SO_2 was initially coordinated to one of the available C=O groups, to two C=O groups from two adjacent layers, or to the S atom from the thiophene units. Only one shows energetically favorable binding with binding energy of –19 kJ/mol. The SO_2 is bound to the carbonyl O atom with S-O (IT unit) distance of 2.7 Å. All the other structures converged to energetically unfavorable states with the binding energy of SO2 from +9 to +95

kJ/mol. This implies that electron transfer occurs between the SO_2 and the IT unit. Given the strong electron-deficient nature of the IT unit, electrons are likely transferred from SO_2 to the IT unit, resulting in the reduction of the IT unit while oxidizing the SO_2 to SO_3.

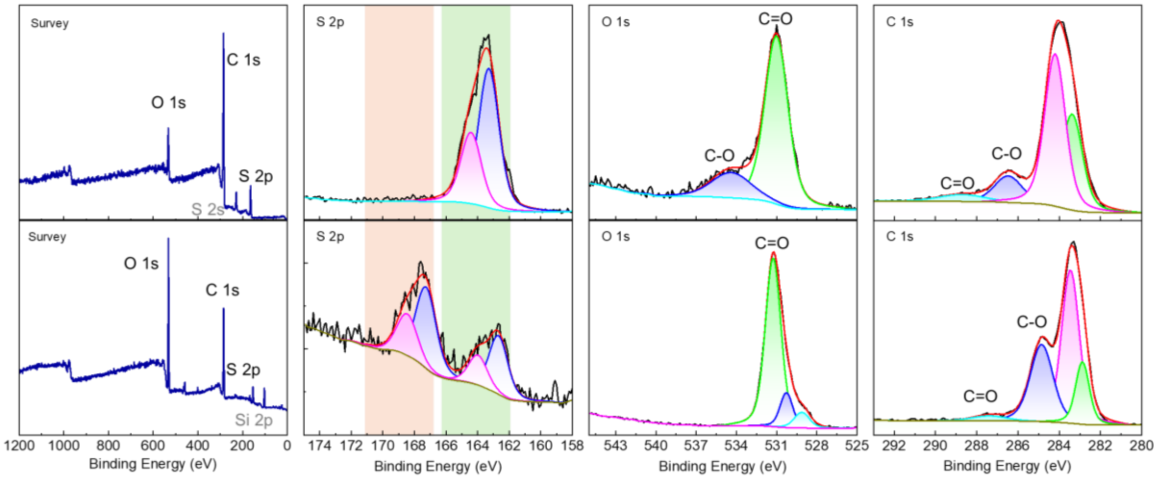

Figure S25. XPS survey spectra and high resolution XPS spectra of S 2p, O 1s and C 1s peaks before (top) and after (bottom) SO_2 sensing.

Analysis of the S 2p XPS peaks clearly reveals the formation of new species with high binding energy ($2p_{1/2}$: 168.5 eV, $2p_{3/2}$: 167.3 eV) on the material's surface. These species can be attributed to adsorbed SO_3, as supported by previous studies.[22–24] (*J. Mater. Chem. A*, **2015**, *3*, 14334–14347; *J. Electrochem. Soc.*, **2020**, *167*, 130507; *J. Mater. Chem. B*, **2020**, *8*, 10788–10796).

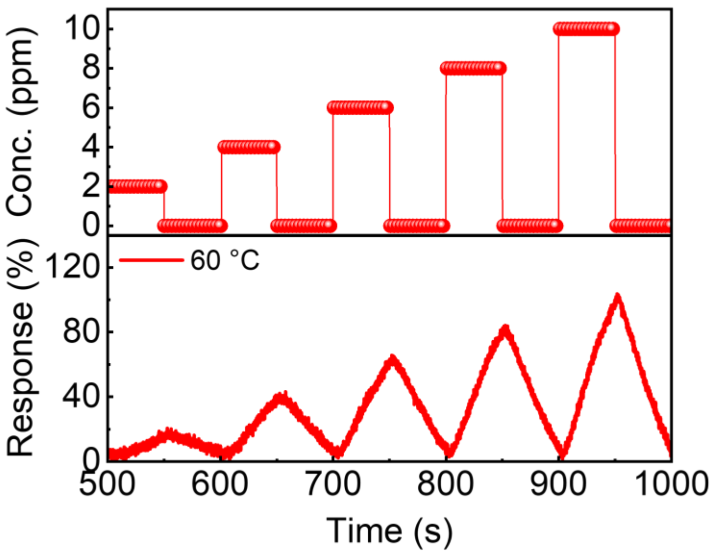

Figure S26. Response-recovery curve of **2DPAV-TBDT-IT** at 60 °C (SO_2 concentrations: 2, 4, 6, 8, 10 ppm).

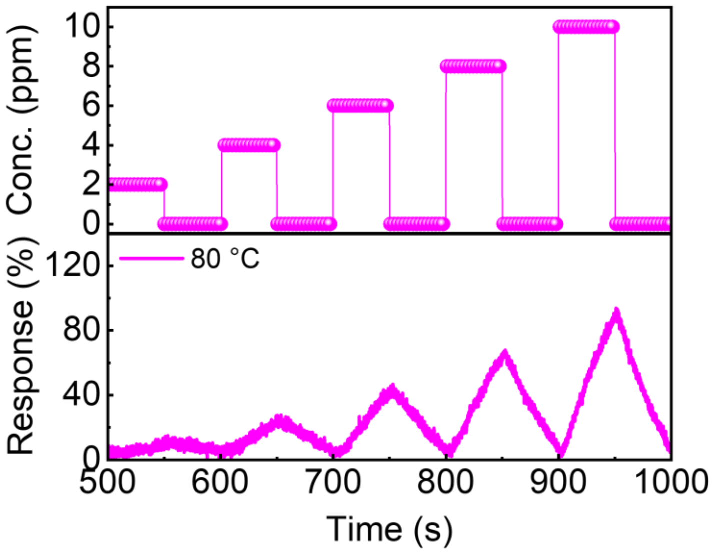

Figure S27. Response-recovery curve of **2DPAV-TBDT-IT** at 80 °C (SO_2 concentrations: 2, 4, 6, 8, 10 ppm).

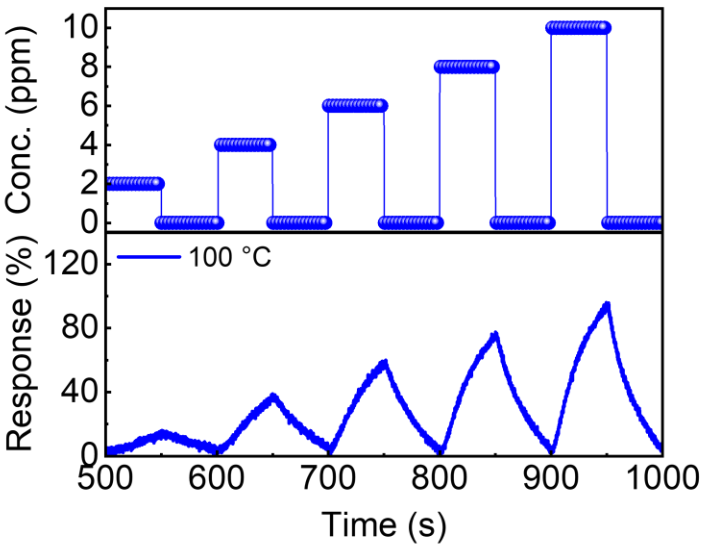

Figure S28. Response-recovery curve of **2DPAV-TBDT-IT** at 100 °C (SO_2 concentrations: 2, 4, 6, 8, 10 ppm).

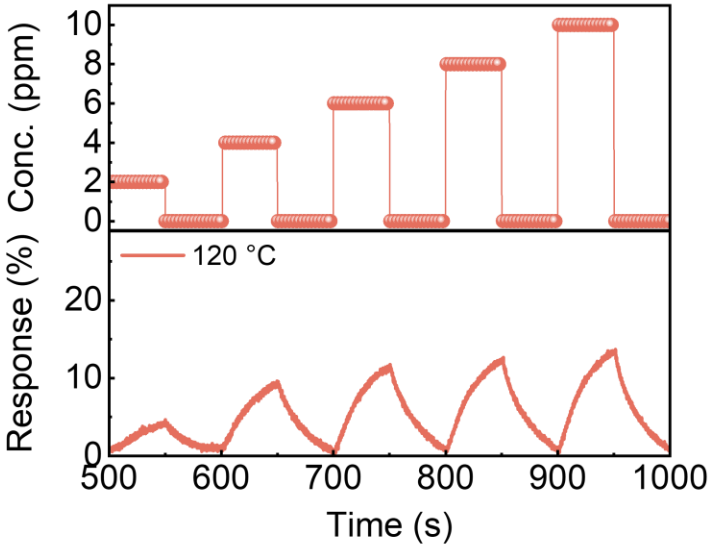

Figure S29. Response-recovery curve of **2DPAV-TBDT-IT** at 120 °C (SO_2 concentrations: 2, 4, 6, 8, 10 ppm).

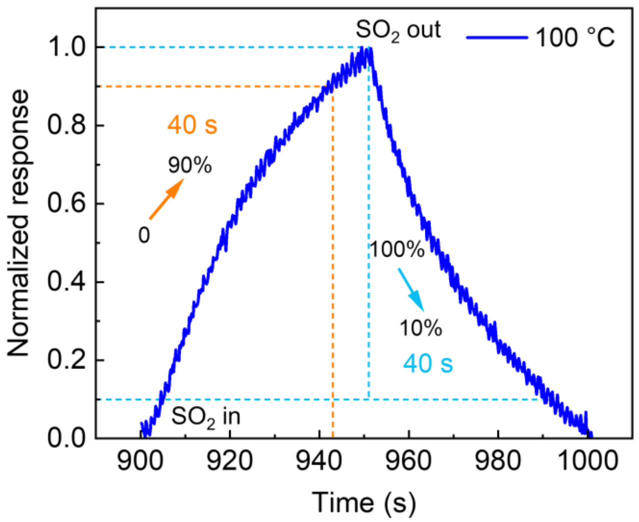

Figure S30. Representative response-recovery curve of **2DPAV-TBDT-IT** at 100 °C (SO_2 concentration: 10 ppm).

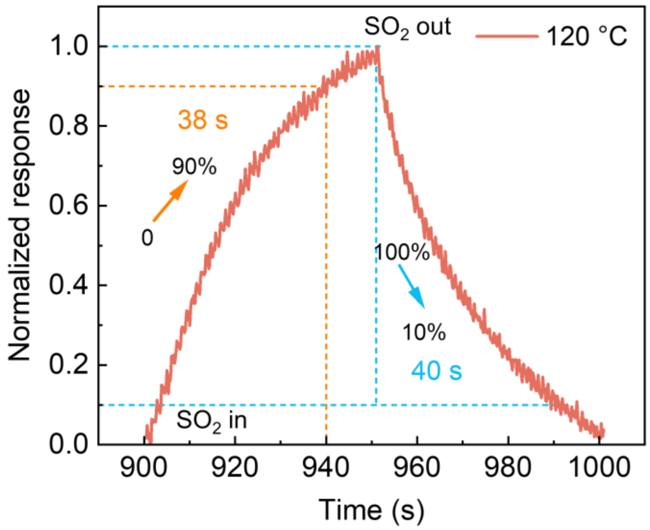

Figure S31. Response-recovery curves of **2DPAV-TBDT-IT** at 120 °C (SO_2 concentration: 10 ppm).

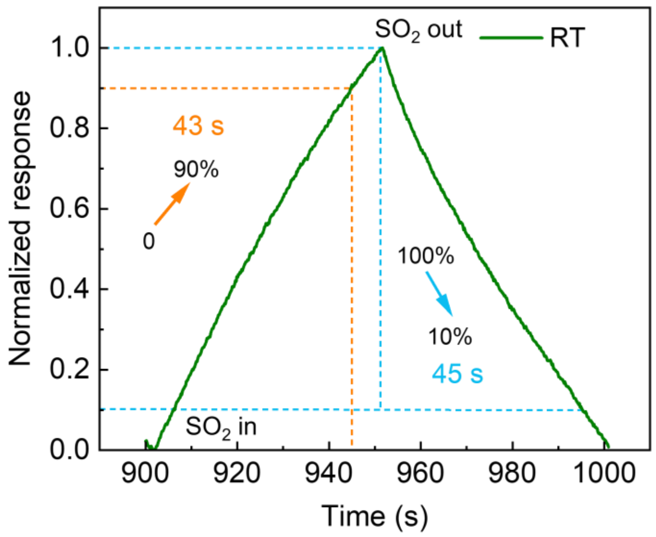

Figure S32. Response-recovery curve of **2DPAV-TBDT-IT** at room temperature (SO_2 concentration: 10 ppm).

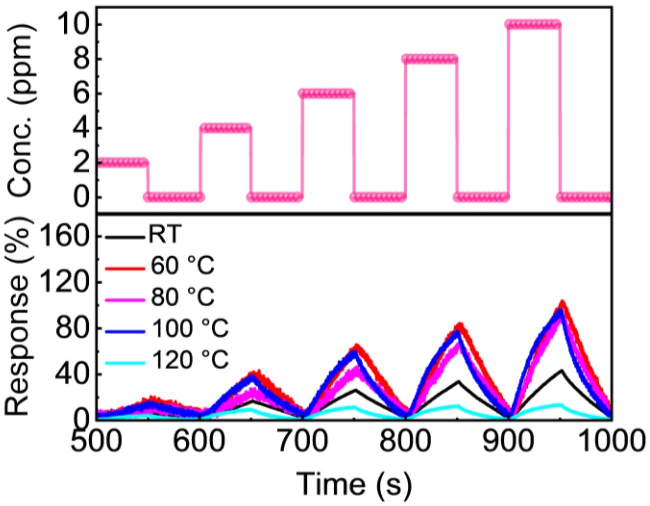

Figure S33. Comparison of response and recovery curves of **2DPAV-TBDT-IT** at different temperatures (RT, 60, 80, 100, and 120 °C) exposure to different SO_2 concentrations (2, 4, 6, 8, and 10 ppm).

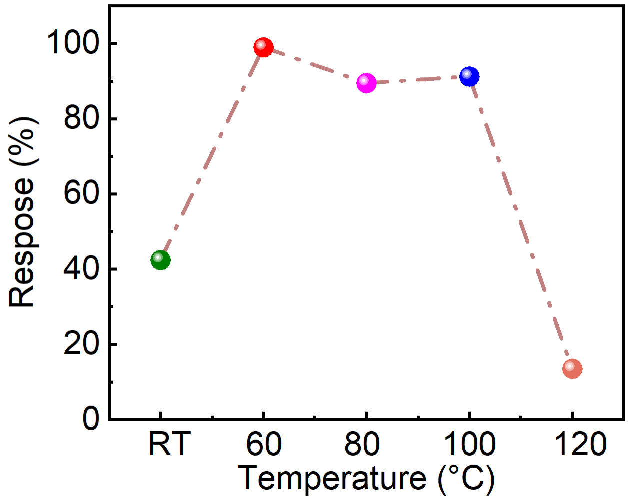

Figure S34. The comparison of the response (%) of **2DPAV-TBDT-IT** at different operating temperatures (RT, 60 °C, 80 °C, 100 °C, and 120 °C). The dashed line was applied to guide the eyes. (SO_2 concentration: 10 ppm). Considering both response/recovery time and response values, working at 100 °C gives the best sensing performance. Further increasing the temperature to 120 °C led to a sacrificed response value (14% at 10 ppm SO_2 concentration).

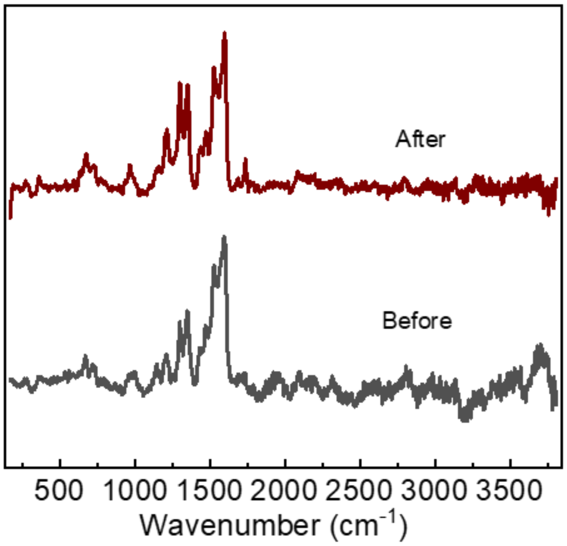

Figure S35. Raman spectra before and after SO_2 sensing cycles. No significant changes were detected, indicating the excellent stability of the **2DPAV-TBDT-IT** material.

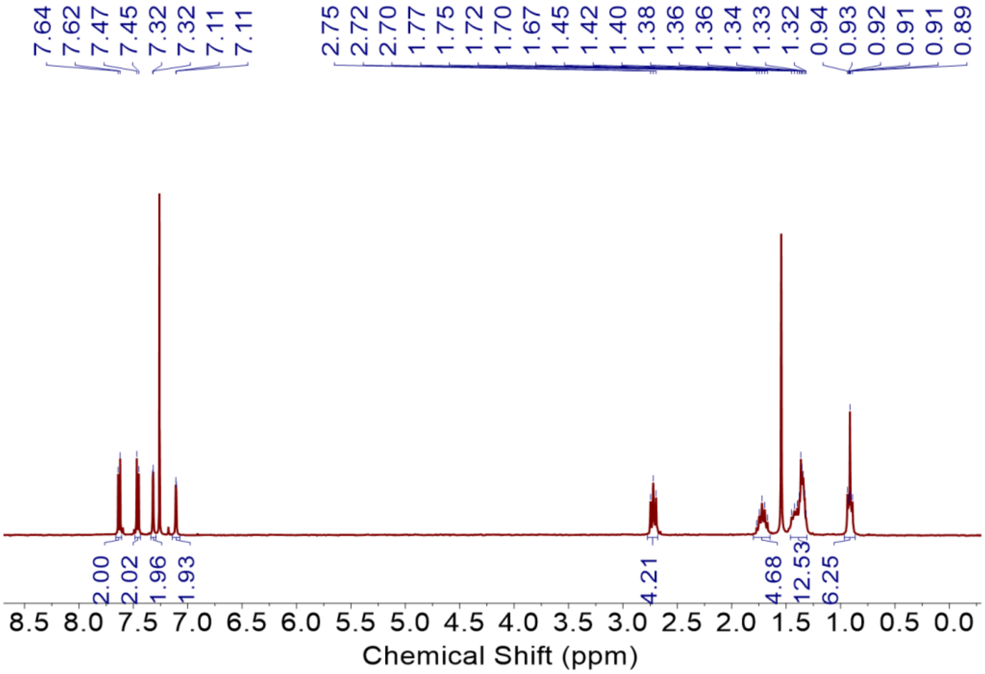

Figure S36. ^{1}H NMR ($CDCl_3$, 300 MHz, 25 °C) spectrum of **TBDT**.

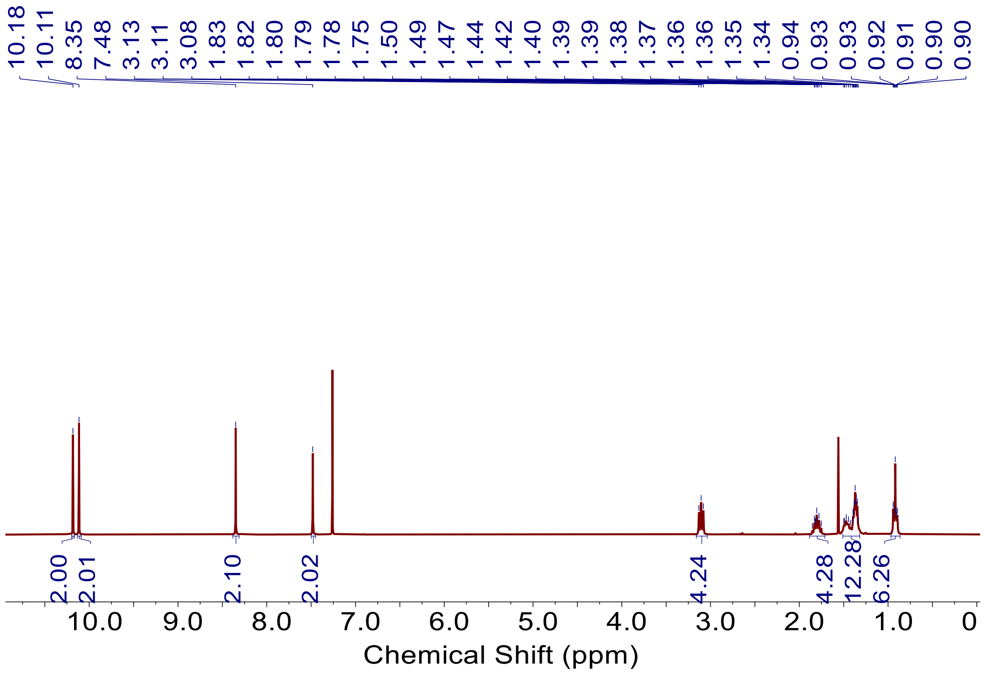

Figure S37. ^{1}H NMR ($CDCl_3$, 300 MHz, 25 °C) spectrum of **m-TBDT**.

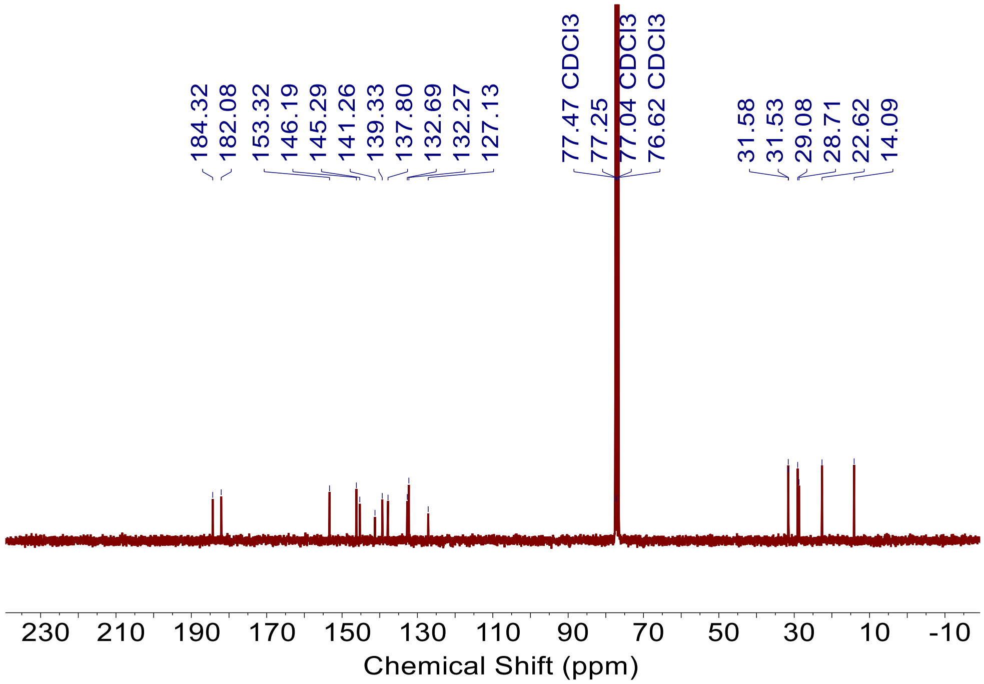

Figure S38. ^{13}C NMR ($CDCl_3$, 75 MHz, 25 °C) spectrum of **m-TBDT**.

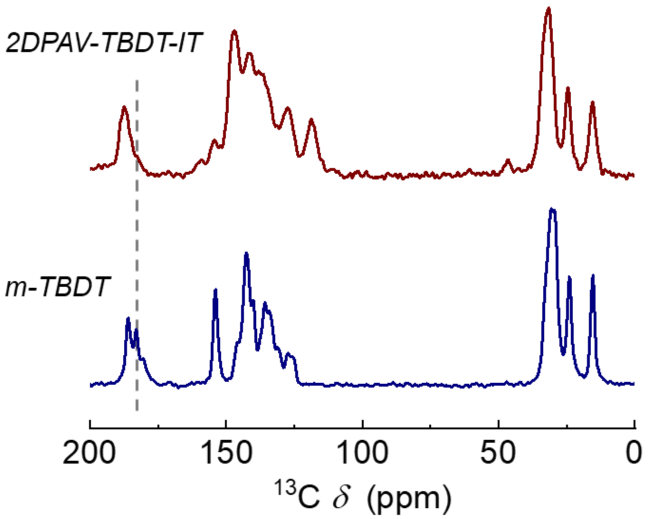

Figure S39. ^{13}C-CP MAS NMR spectra of **m-TBDT** and **2DPAV-TBDT-IT**.

Section D. Supporting Tables

Table S1. Condition screening for synthesis of crystalline **2DPAV-TBDT-IT**.

Entry No.	Solvent/Catalyst[a]	Reaction Temperature[b] (°C)	Crystallinity
1	DMAc/6 M AcOH (50:1)	120	amorphous
2	DMAc/0.1 M *p*TSA	120	amorphous
3	Acetic anhydride	120	amorphous
4	Methanol/NaOH (5 eq.)[a]	80	amorphous
5	DMAc/NaOH (5 eq.)[a]	120	amorphous
6	Mesitylene/6 M AcOH (100:1)	120	High
7	Mesitylene/Dioxane/6 M AcOH (50:50:1)	120	Low
8	Mesitylene/TFA (50:1)	120	Low
9	Mesitylene/Propionic acid (100:1)	120	High
10	Mesitylene/Acetic anhydride (100:1)	120	Low
11	DMAc/*o*DCB/Pyridine (10:10:3)	100	Low

[a]The reaction concentration was set to 0.02 M for m-TBDT, and the equivalent of the catalyst was calculated based on the m-TBDT monomer.

[b]The reaction time was kept at 72 hours.

Table S2. Comparison of sensing performance of **2DPAV-TBDT-IT** with reported sensors.

Sample	LOD (ppb)	Response time (s)	Recovery time (s)	Ref.
**2D c-COFs**				
**2DPAV-TBDT-IT**	0.088	40	40	*This work*
NKCOF-12	86	208	242	J. Am. Chem. Soc., 2024, 146, 33509−33517
**MOFs**				
Zr-MOF	5	435	185	ChemNanoMat, 2021, 7, 1117–1124
FM-300 MOF	5	1000	500	J. Mater. Chem. A, 2018, 6,5550–5554
Ni_3HHTP_2 MOF	625	13	32	Appl. Phys. A, 2020, 126, 1-9
NRs-Ni_3HHTP_2 MOF	625000	20	23	J. Mater. Sci.: Mater. Electron., 2021, 32, 18657–18668
**MOF composites**				
Zn-BDC@rGO	200000	60	120	Appl. Phys. A., 2023, 129, 828
UiO-66-NH_2/PAN NM	100	235	300	Appl. Surf. Sci., 2023, 613, 155772
PAN@UiO-66-NH_2 NM	1000	255	170	Chem. Eng. J., 2022, 428, 131720
MOF-ZIF-67/CNTs	500	78	32	J. Mater. Chem. A,, 2018, 6,12115–12124
MOFs-TiO2/rGO	250	100	200	J. Mater. Sci.: Mater. Electron., 2019, 30, 11070–11078
$ZnFe_2O_4$/rGO	1000	46	54	Rare Metals., 2021, 40, 1604–1613

Table S3. Starting materials and resources

Chemical Name	Resource
Benzo[1,2-b:4,5-b']dithiophene-4,8-dione, 98%	TCI Deutschland GmbH
n-butyllithium solution (2.5 M in hexanes)	TCI Deutschland GmbH
s-indacene-1,3,5,7(2H,6H)-tetraone, 99%	BLD Pharmatech GmbH
Mesitylene, 99%	Acros
1,4-Dioxane, 99%	Acros
Anhydrous tetrahydrofuran, 99.8%	Alfa Aesar
Anhydrous acetone, 99.5%	Alfa Aesar
Acetic acid, 99%	Sigma-Aldrich
NH_4OH, 99%	Sigma-Aldrich
1,3-Dimethyl-2-imidazolidinone (DMI), anhydrous, 99%	Sigma-Aldrich
N,N-Dimethylacetamide (DMAc), anhydrous, 99.5%	Alfa Aesar
1-Methyl-2-pyrrolidinone (NMP), 99%	Sigma-Aldrich

Section E. Supporting References

[1] Khan, A. H.; Peikert, K.; Hoffmann, F.; Fröba, M.; Bertmer, M. *J. Phys. Chem. C* **2019**, *123*, 4299–4307.

[2] Li, J., Lu, Y., Ye, Q., Cinke, M., Han, J., & Meyyappan, M. (2003). Carbon nanotube sensors for gas and organic vapor detection. Nano letters, 3(7), 929-933. https://doi.org/10.1021/nl034220x

[3] Currie, L. (1995). Nomenclature in evaluation of analytical methods including detection and quantification capabilities (IUPAC Recommendations 1995). Pure and Applied Chemistry, 67(10), 1699-1723. https://doi.org/10.1351/pac199567101699

[4] Gaus, M.; Goez, A.; Elstner, M. *J. Chem. Theory Comput.* **2013**, *9*, 338–354.

[5] Kresse, G.; Furthmüller, J. *Comput. Mater. Sci.* **1996**, *6*, 15–50.

[6] Kresse, G.; Furthmüller, J. *Phys. Rev. B* **1996**, *54*, 11169–11186.

[7] Blöchl, P. E. *Phys. Rev. B* **1994**, *50*, 17953–17979.

[8] Kresse, G.; Joubert, D. *Phys. Rev. B* **1999**, *59*, 1758–1775.

[9] Perdew, J. P.; Burke, K.; Ernzerhof, M. *Phys. Rev. Lett.* **1996**, *77*, 3865–3868.

[10] Krukau, A. V.; Vydrov, O. A.; Izmaylov, A. F.; Scuseria, G. E. *J. Chem. Phys.* **2006**, *125*, 224106.

[11] Monkhorst, H. J.; Pack, J. D. *Phys. Rev. B* **1976**, *13*, 5188–5192.

[12] Grimme, S. *J. Comput. Chem.* **2006**, *27*, 1787.

[13] Becke, D. *J. Chem. Phys.* **1993**, *98*, 5648–5652.

[14] Lee, C.; Yang, W.; Parr, R. G. *Phys. Rev. B.* **1988**, *37*, 785.

[15] Vosko, H.; Wilk, L.; Nusair, M. *Can. J. Phys.* **1980**, *58*, 1200–1211.

[16] Stephens, P.J.; Devlin, F.J.; Chabalowski, C.F.; Frisch, M.J. *J. Phys. Chem.* **1994**, *98*, 11623–11627.

[17] Weigend, F.; Ahlrichs, R. *Phys. Chem. Chem. Phys.* **2005**, *7*, 3297.

[18] Stoychev, G. L.; Auer, A. A.; Neese, F. J. *Chem. Theory Comput.* **2017**, *13*, 554–562.

[19] Liu, Y. M.; Zhang, H.; Yu, H. Liao, Z.; Paasch, S.; Xu, S.; Zhao, R.; Brunner, E.; Bonn, M.; Wang, H.; Heine, T.; Wang, M.; Mai, Y.; Feng, X. *Angew. Chem. Int. Ed.* **2023**, *62*, e202305978.

[20] Xu, H.; Li, J.; Li, P.; Shi, J.; Gao, X.; Luo, W. *ACS Appl. Mater. Interfaces* **2021**, *13*, 49194–49205.

[21] Wang, S.; Fu, Y.; Wang, F.; Wang, X.; Yang, Y.; Wang, M.; Wang, J.; Lin, E.; Ma, H.; Chen, Y.; Cheng, P.; Zhang, Z. *J. Am. Chem. Soc.*, **2024**, *146*, 33509−33517.

[22] Favaro, M.; Carraro, F.; Cattelan, M.; Colazzo, L.; Durante, C.; Sambi, M.; Gennaro, A.; Agnoli, S.; Granozzi, G. *J. Mater. Chem. A*, **2015**, *3*, 14334–14347.

[23] Sicklinger, J.; Beyer, H.; Hartmann, L.; Riewald, F.; Sedlmeier, C.; Gasteiger H.A. *J. Electrochem. Soc*., **2020**, *167*, 130507.

[24] Yang, X.; Guo, Y.; Liang, S.; Hou, S.; Chu, T.; Ma, J.; Chen, X.; Zhou, J.; Sun, R. *J. Mater. Chem. B*, **2020**, *8*, 10788–10796.
